# Clinical Pharmacology

**Published:** 2008-10

**001**

**‘Fixing’ the FDCS: We need to do it now**

Singh J, Gautam CS, Sandhu G

**Government Medical College and Hospital, Sector-32, Chandigarh - 160 030, India.**

**Introduction:**

Indian pharmaceutical market is flooded with countless fixed dose combinations (FDCs). Senseless combination of drugs is risky and unethical in a country like ours where virtually any drug is ‘over the counter.’

**Methods:**

To get a glimpse of the countless FDCs available in India we looked into all FDCs of five drugs - three highly efficacious - Atorvastatin, Amlodipine, Amoxycillin and two with question on safety issues, yet highly prescribed in India - Nimesulide and Rosiglitazone (CIMS July - Oct 2007) and analyzed these FDCs for rationality.

**Results:**

A total of 47 FDCs were listed. Atorvastatin had 09 (19.14%) FDCs, Amlodipine 08 (17.02%), Amoxycillin 12 (25.53%), Nimesulide 13 (27.65%) and Rosiglitazone 5 (10.63%) FDCs. Rationality analysis revealed shockingly few rational FDCs - 1/9 (11.01%) for Atorvastatin, 3/8 (37.05%) for Amlodipine, 1/12 (8.33%) for Amoxycillin and none for Rosiglitazone and Nimesulide. Overall 89.36% (42/47) FDCs were deemed irrational and only 10.63% (5/47) FDCs were rational.

**Discussion:**

That 89.36% FDCs are irrational gives enough idea of the status of innumerable FDCs dumped in the market. This representative analysis reveals a ground reality - Indian pharma industry thrives on irrational FDCs. Irrational FDCs not only cause economic burden on patients, can lead to ADRs and foster careless prescribing attitude. The lure of FDCs has entangled even ‘research-based’ companies. It is our duty as medical teachers to instill the concept of rational drug use in students and train the prescribers to shun irrational combinations. Further work to limit the glut of irrational FDCs is required.

**Conclusions:**

Its high time legal enactment regarding irrational FDCs be implemented in India to protect unsuspecting patients from the gullible ones. Prescribers should refrain from putting their patients on dubious combinations and help in ‘fixing’ irrational FDCs.

**002**

**Effect of noni fruit juice on lipid profile in diabetic patients**

Sabitha P^1^, Adhikari PM^1^, Kamath A^2^

**^1^Kasturba Medical College, Mangalore; ^2^Kasturba Medical College, Manipal, India.**

**Introduction:**

Noni (*Morinda citrifolia L*) fruit juice [NFJ] is used for atherosclerosis and diabetes in alternative medicine. This randomized placebo controlled double-blind study aimed at investigating the lipid lowering efficacy of NFJ in diabetics.

**Methods:**

34 diabetic patients were enrolled into the study. After measuring fasting plasma lipid profile [LP], subjects were randomized into two groups, either to receive NFJ or placebo at a dose of 15mL, twice daily for 21 days. On day 22, LP was repeated. The mean changes in LP from baseline were calculated in each group and were compared between groups using repeated measures ANOVA. *P*<0.05 was considered statistically significant.

**Results:**

Mean age of patients [63 years] and mean baseline LP measures were comparable between two groups. Mean changes in LP measured in each group were insignificant [mean±SEM of total cholesterol (192.3±21.9 - 196.5±30.8 and 188.9±26.6 -185.8±19.9); LDL cholesterol (123.9±16.6 - 123.3±12.3 and 124.5±19.3 - 122.7±18.1); HDL cholesterol (47±5.2 - 46.2±4.8 and 45.8±6.7- 45.3±5); triglycerides (113±32.4 - 108.7±25.1 and 100±23.4 - 96±19.3) in NFJ and placebo treated groups, respectively] and were comparable between groups. Normal hematological, liver and renal function tests measured at the end of study indicated the safety of NFJ consumption.

**Conclusion:**

Present study that was carried out in a small population of diabetic patients measuring LP within normal range, failed to demonstrate the lipid lowering efficacy of NFJ on short term use. Long-term studies with large sample size need to be carried out to explore the therapeutic potential of NFJ in hyperlipidemia.

**003**

**A comparative study of efficacy of inhaled aminoglycoside tobaramycin in COPD patients**

Sharma KK, Sharma L

**SMS Medical College Jaipur, India.**

**Objective:**

To evaluate the efficacy of inhaled aminoglycoside tobramycin in COPD patients.

**Methods:**

This study was conducted on patients suffering from COPD with frequent chest infection since last 10 years. In this study indoor patients of 50-70 years were selected and divided into two groups of fifty of each this study was done in private hospital at Jaipur.

**Group 1:**

Patient were treated by with conventional Bronchodilator like beta agonists and anticholinergics. In addition to oxygen therapy and oral antibiotics.

**Groups 2:**

Patients were treated with nebulisation with tobramycin 300mg twice daily. In addition to conventional Bronchodilator oxygen and similar oral antibiotics. For symptomatic relief other drugs like paracetamol, proton pump inhibitor antiemetics were also prescribed. The progression of treatment was monitored by serial estimation of total leucocytes count, ESR chest x-ray, Quantity of sputum production daily, Pulmonary function test on alternate day, oxygen saturation by pulse oxymeter, Arterial blood gas analysis, clinical parameter like respiratory rate, blood pressure, fever, patient wellbeing.

**Results:**

This study showed that group -2 patients showed improved out come in terms of less duration of hospital stay (5 days in group-2 in compared 7 days in group-1). Decreased quantity of sputum production, early clearance of chest X-ray, Saturation >92%, Rapid normalization of cell counts, Patient wellbeing in comparison to group -1 patient. Pulmonary function test showed rapid improvement in functions like increase in FEV-1 in Group-2 patients showed less frequency of hospital admission.

**Conclusion:**

Inhaled aminoglycoside tobramycin therapy showed significant improvement in COPD patient in terms of less duration of hospital stay and improvement in pulmonary function tests.

**004**

**Study of reservoir effect of topical corticosteroid in an experimental animal model**

Abidi A, Ahmad F, Kumar A, Singh SK

**J.N.Medical College, A.M.U Aligarh, India.**

**Introduction:**

Topical corticosteroids, the most commonly used preparations in the treatment of skin diseases have potent vasoconstrictive, antiproliferative, anti-inflammatory and immunosuppressive actions. But there usage several times a day led to an increased risk of side effects. Hence the aim of this study was to demonstrate a reservoir of topically applied corticosteroid (Clobetasol propionate) experimentally in rabbits as rabbit skin is akin to human skin so that one can maximize their efficacy and safety as therapeutic agents.

**Methods:**

The work was carried out on albino rabbits using the topical steroid Clobetasol propionate 0.05% cream. It was applied on the back of rabbit and after 1 hour occlusion histamine induced wheal suppression test was performed and wheal area measured at 10 mins till day 7.

**Results:**

Maximum wheal suppression was seen on day 1(p value <.001). Interday comparison of mean wheal size showed no significant difference (p value >.05) on day 2, 3 and 4 as compared to day 1. Day 5-7 showed highly significant difference (p value <.001) as compared to day 1, thereby suggesting that the reservoir effect of topical corticosteroid persisted till day 4.

**Conclusions:**

This work demonstrated that histamine induced wheal by the topical steroid, Clobetasol propionate was suppressed till day 4, suggesting that the reservoir persisted till day 4. Due to this depot effect, we can advocate less frequent application of topical corticosteroid hence reducing the cost of therapy and adverse effects of corticosteroid and can also improve patient compliance.

**005**

**Clinical evaluation of two antiemetic combinations palonosetron dexamethasone versus ondansetron dexamethasone in chemotherapy of head and neck cancer**

Kaushal J, Gupta MC, Kaushal V, Dhankhar R, Atri R, Bhutani G, Verma S

**Pt. B.D. Sharma Post Graduate Institute of Medical Sciences Rohtak Haryana India.**

Palonosetron and ondansetron are two selective 5ht3 antagonist that have shown remarkable efficacy in controlling nausea and vomiting following administration of highly emetic anticancer chemotherapy, the efficacy of these is known to be enhanced by concurrent administration of dexamethasone. A randomized, crossover trial was conducted on 30 head and neck cancer patients, receiving cancer chemotherapy, comparing the antiemetic efficacy of palonosetron plus dexamethasone (pd) schedule versus ondansetron plus dexamethasone (od) schedule. The combination chemotherapy schedule consisted of docetaxel 60mg/m^2^, carboplatin 300mg/m^2^ and 5fu 600mg/m^2^. The patients were divided into two groups. One group was given pd schedule (inj palonosetron 0.25 Mg plus inj dexamethasone 16 mg intravenously half an before chemotherapy) and the other group od schedule (inj ondansetron 16 mg plus inj dexamethasone 16 mg intravenously half an before chemotherapy) in the first cycle. For the subsequent cycle, crossover of the antiemetic schedules was done. The antiemetic effects were evaluated by recording frequency of vomiting, and; acute and delayed frequency, intensity and duration of nausea. Complete response (no emesis and no rescue medication) during the acute (0-24 hours) interval post therapy was 25/30 (83.3%) In pd schedule and 24/30 (80%) in od schedule. Delayed nausea was reported by 11/30 (36.6%) Patients in pd schedule and 13/30 (39.4%) Patients in the od schedule. The mean duration of delayed nausea was 26 hrs with pd schedule and 32 hours with od schedule. The results suggest that palonosetron plus dexamethasone (pd) schedule was slightly better than ondansetron plus dexamethasone (od) schedule for control of emesis in cancer chemotherapy, though this difference was not statistically significant.

**006**

**Effect of atorvastatin and pioglitazone on endothelial dysfunction in non-diabetic patients with hypertension or stable angina**

Pattan V, Maulik SK, Seth S, Gupta YK

**All India Institute of Medical Sciences, New Delhi, India.**

**Aim:**

Endothelial dysfunction is a key pathological factor in hypertension and stable angina. Both statins and PPAR-gamma agonists exert pleiotropic effects and improve endothelial dysfunction. The aim of the present study is to evaluate the effect of atorvastatin, pioglitazone, and compare the individual effects with their combination in non-diabetic patients with hypertension or stable angina.

**Methods:**

Inclusion criteria: Patients with stable angina and/or hypertension between 35-65 years belonging to both sexes.

**Exclusion criteria:**

Diabetes mellitus, hepatic/renal dysfunction, pregnant or lactating women, tobacco consumption within previous 6 months, CHF [NYHA class II TO IV], recent myocardial infarction, history of unstable angina, history of allergy to statins or pioglitazone, severe or uncontrolled hypertension(>160/110 mmHg). The patients were randomly assigned to 3 treatment arms-Group1: pioglitazone (15mg OD) + atorvastatin (10mg OD); Group2: pioglitazone (30mg OD) alone; Group3: Atorvastatin (10mg OD) alone. The midterm interim analysis was done after enrolling 13, 8 and 9 patients in groups 1, 2 and 3 respectively using biochemical markers (hsCRP, sICAM-1 and sVCAM-1 using human ELISA kits) of endothelial dysfunction before and after 12 weeks of administration of drugs.

**Results:**

Both atorvastatin and pioglitazone monotherapy showed significant (*P*<0.05) reduction in biochemical markers of endothelial dysfunction. The combination therapy showed the synergistic effect (*P*<0.05) on biochemical markers of endothelial dysfunction.

**Conclusion:**

The study is suggestive of therapeutic advantage of combining atorvastatin and pioglitazone. However the final analysis is required to arrive at a meaningful conclusion.

**007**

**Study of oral propranolol for preoperative anxiolysis in adults**

Khare A, Khadke SV, Subedar S, Khadke VV, Patil AW

**Shri Bhausaheb Hire Govt. Medical College And Hospital, Dhule, India.**

**Introduction:**

Large numbers of patients undergo surgical interventions and most of them have high levels of anxiety and stress before surgery. This study was conducted to assess and compare anxiolytic effects of two doses of oral propranolol given in premedication.

**Methods:**

In this double blind, randomized, prospective, case controlled study, 60 healthy patients undergoing minor elective surgery were studied. Patients received either Propranolol 20 mg (n = 20), Propranolol 40 mg (n = 20), orally with sips of water 2 hr prior to proposed time of surgery or no anxiolytic premedication (n=20).

**Results:**

Using Student’s paired‘t’ test and Wilcoxon signed rank test, significant difference was observed in preoperative and postoperative anxiety, pulse rate and systolic blood pressure, among the 20 mg and 40 mg group. No significant side effects were observed.

**Conclusions:**

Both 20 mg and 40 mg doses are effective for preoperative anxiolysis but 20 mg dose seems to be better. It gives significant reduction in anxiety with minimal side effects. No measurable significant side effects were seen with the use of this drug in the dosages used in this study. Thus we recommend the use of oral Propranolol in given doses for reducing anxiety in preoperative as well as postoperative period.

**008**

**Comparative study of nebivolol and (S) - atenolol on blood pressure and heart rate in essential hypertensive patients**

Sahana GN, Sarala N, Kumar TN, Lakshmaiah V

**Sri Devaraj Urs Medical College, Kolar, Karnataka, India.**

**Introduction:**

Hypertension is a major problem leading to morbidity and mortality. Essential hypertension is a condition where cause for rise in blood pressure is unknown.

**Objective:**

To study the effect of Nebivolol 5mg versus (S)-Atenolol 25mg in essential hypertension.

**Methods:**

The study was conducted at RLJH and Research Centre which included 30 patients in each group with essential hypertension. Patient sex, age, presenting illness and family history were recorded. Investigations like blood sugar, urine analysis, kidney function test, lipid profile and ECG were done before starting the treatment. Any adverse effects during the treatment were noted. Blood pressure and heart rate were recorded at baseline and during follow up. One group received Nebivolol 5mg once daily and other group (S)-Atenolol 25 mg once daily. Patients were followed up every 15 days for 3 months.

**Results:**

Nebivolol group had 18 males and 12 females with mean age 50.6±9.5yrs, (S)-Atenolol had 16 males 14 females with mean age 54.4±9yrs. Patients receiving Nebivolol and (S)-Atenolol showed a significant fall (*P*<.0001) in systolic, diastolic blood pressure and heart rate at the end of 1st, 2nd and 3rd month when compared to baseline. The fall in systolic and diastolic blood pressure was insignificant between the groups, but fall in heart rate was significant (*P*<.0001) with (S)-Atenolol compared to Nebivolol. Adverse effects like headache, dizziness and fatigue were reported with both drugs.

**Conclusion:**

Reduction of blood pressure with Nebivolol and (S)- Atenolol was similar, but fall in blood pressure from baseline was highly significant in both groups.

**009**

**Therapeutic drug monitoring by high performance liquid chromatography - in cancer chemotherapy using methotrexate**

Kaleekal T, Bakhshi S, Gupta YK

**All India Institute of Medical Sciences, New Delhi, India.**

Methotrexate(MTX) is a folic acid antagonist blocks an enzyme needed by the cell to live, known as antimetabolites. It inhibits the synthesis of DNA, RNA, thymidylate, and protein. This interferes with the growth of cancer cells which should be destroyed. Methotrexate is used as an antineoplastic in the treatment of a wide variety of malignancies. Methotrexate clearance rates vary widely and are generally decreased at higher doses. Delayed drug clearance has been identified as one of the major factors responsible for methotrexate toxicity. Plasma concentrations of MTX >1*µ*m/L at approximately 42 hours have been associated with increased risk of toxicity despite increased leucovorin rescue. Threfore, Therapeutic Drug Monitoring(TDM) has an important role in the cancer chemotherapy using Methotrxate. High Performance Liquid Chromatography (HPLC) is used to monitor the plasma concentration of Methotrexate. Blood samples of patients treated with Methotrexate at the Rotary Cancer Hospital of our Institute is being screened for MTX levels. Therapeutic Drug Monitoring of MTX is found to be useful in leucovorin dose adjustments in patients of HDM administration. Blood level is monitored up to 72 hours or till the level reaches >0.2*µ*m/L. Blood sample is taken in an EDTA vial and plasma is separated. Agilent HPLC 1200 series with Chemstation software is in use. RP 8 Column is used. Methotrexate is estimated at 307nm using a UV detector. Concentration of Methotrexate in blood is calculated from the area of the chromatogram corresponding to the concentration using calibration curve. Reproducibility and linearity within a range from 0.250 to 16mg/L of MTX is observed.

**010**

**Effects of simultaneous administration of cypermethrin and chlorpyriphos on pharmacokinetics and biochemical profiles in mice**

Paul S^1^, Kundu T^1^, Dutta S^1^, Khargharia S^2^ Mandal T K^2^ Chakraborty A^2^

**^1^Medical College, Kolkata; ^2^West Bengal University of Animal and Fisheries Sciences, West Bengal, India.**

**Objective:**

To evaluate the blood and tissue concentration as well as biochemical parameters after administration of commonly used pesticides: cypermethrin, chlorpyrifos and co-administration of both in mice.

**Materials and Methods:**

Swiss albino mice of either sex were divided into 4 groups (n=18) Group II received Cypermethrin (25 mg/kg), group III received chlorpyrifos (6mg/kg) and group IV received both cypermethrin (25 mg/kg) and chlorpyrifos (6mg/kg) orally daily for 21 days. Group I was considered as control. Six animals from each group were sacrificed on 7, 14 and 21 days post dosing and blood samples and tissues (muscle, liver, heart, brain and kidney) were collected. Concentration of both cypermethrin and chlorpyrifos was measured (*µ*g ml^-1^) and biochemical parameters were analyzed in all the blood samples. The amount of cypermethrin and chlorpyrifos (*µ*g gm^-1^) was estimated in individual tissues. A mean of six replicates were taken and data analyzed statistically.

**Results:**

Both cypermethrin and chlorpyrifos were recovered from blood and different tissue samples from 7th day onwards. Their concentration was found to be less when co administered in comparison to individual dosing at the end of study. Highest concentration was observed in the liver in all the treated groups. AST, ALT and blood glucose levels were increased considerably in treated groups while protein level was decreased.

**Conclusions:**

The results indicate that concomitant administration of both cypermethrin and chlorpyrifos may have some pharmacokinetic type of drug interaction which influences both biochemical parameters as well as tissue concentrations favorably.

**011**

**Prophylactic use of antimicrobials in hysterectomy**

Heethal JP, Sarala N, Kumar TN, Hemalatha MS

**Sri Devaraj Urs Medical College, Kolar, Karnataka, India.**

**Introduction:**

Antimicrobial prophylaxis has been common in surgical field. Antimicrobial prophylaxis in gynaecological surgeries decrease morbidity and mortality.

**Objective:**

To analyse prophylactic use of antimicrobials in hysterectomy and the frequency of post operative complications.

**Methods:**

A prospective study including seventy five patients undergoing hysterectomy admitted to R L J hospital and research centre, Kolar was undertaken. Patient’s age, socioeconomic status, Hb% and indication for sugery was noted. Antimicrobial used, dosage schedule, timing of administration was recorded. Pus, blood and urine of patients with post operative complications were subjected to culture and sensitivity.

**Results:**

The mean age of the patients was 42.6±7.6. Thirty two patients were of low socioeconomic status with Hb% of 8.9±1.3g%. Fibroid uterus was the common indication for the surgery. Antimicrobials used were ceftriaxone, cefotaxime and metronidazole. Use of two drug(84%) or three drug combinations(16%) were common which included third generation cephalosporins with metronidazole, and gentamicin was added in three drug combinations. Antimicrobials were administered 1-4 hours before surgery in 61 patients and continued upto 5-8 days. Post operative complications (24/75 patients) like wound infection, urinary tract infection and fever were significantly (*P*<0.05) more in patients who did not receive antimicrobials preoperatively. E coli was the common organism isolated from pus and urine which was resistant to third generation cephalosporins and sensitive to amikacin.

**Conclusions:**

Pre-operative antimicrobial administration ensures therapeutic concentration of the drug during the period of potential contamination. Periodic surveillance of antimicrobial prophylaxis is essential to detect the emergence of resistance.

**012**

**A retrospective analysis of adverse drug reactions in elderly in a tertiary referral centre in Mumbai, India**

Rupawala AH, Kshirsagar NA, Gogtay NJ

**Seth GS Medical College and KEM Hospital, Parel, Mumbai, India.**

**Background:**

Adverse Drug Reactions (ADRs) account for significant morbidity and mortality in elderly. Beers criteria are a set of standard criteria for guiding drug prescription in elderly.

**Objective:**

To estimate the ADR burden in elderly in India and use of Beers criteria for assessing appropriateness of Drug Prescription in them.

**Methods:**

ADR data collected a tertiary referral centre under the National Pharmacovigilance Programme for the years 2005 and 2006 was analysed. Elderly individuals were defined as those aged ≥58 years.

**Results:**

In 2005, 321 ADRs were reported and in 2006 there were 673. Of them elderly constituted 18.91% (60) in 2005 and 11.84% (44) in 2006. About 11.67% (7) of the ADRs in elderly in 2005 were due Beers criteria medications while in 2006 there were none. Two thirds of the ADRs in both years were found to be due to antidiabetics, oral anticoagulants and antiplatelets and narrow therapeutic index drugs. Warfarin, digoxin and insulin accounted for a quarter of the ADRs.

**Conclusions:**

Some commonly used medications account for a major proportion of ADRs in elderly. Prospective studies of similar nature could help further assessment of ADR burden in elderly.

**013**

**Spontaneous adverse drug reaction reporting in oncology unit at Dayanand Medical College and Hospital**

Singh T, Kaur K, Bhagat S, Sekhon JS, Chopra SC, Kaushal S

**Dayanand Medical College and Hospital Ludhiana, India.**

**Introduction:**

Cancer chemotherapy, being a multi-drug therapy is associated with a high incidence of ADRs. Hospital based adverse drug reaction (ADRs) monitoring and reporting programs aim to identify and quantify the risk associated with the use of drugs.

**Methods:**

A prospective, spontaneous ADR reporting study was conducted in 136 oncology ward patients over 7 months. The WHO definition of an ADR was adopted. The chemotherapy, premedication and the treatment of the ADRs was noted. The Naranjo Algorithmic scale was used for causality assessment.

**Results:**

The patients (9-81 years) included 69 females and 67 males. The average number of anticancer drugs used was 2.5/patient. Pyrimidine antagonists was the most frequently prescribed class of drugs followed by platinum-containing compounds, antibiotics, alkylating agents, taxanes and vinca alkaloids. The commonest ADRs were alopecia (7.4%), constipation (5.2%), diarrhea (4.4%), anorexia (4.4%), and neutropenia (4.4%). Alopecia occurred with taxanes, platinumcontaining compounds and vinca alkaloids; constipation with platinum-containing compounds, taxanes and alkylating agents; diarrhea with platinum-containing compounds and pyrimidine antagonists; anorexia with platinum-containing compounds and antibiotic chemotherapeutic agents; neutropenia with platinumcontaining complexes and antibiotic chemotherapeutic agents. Of the 45 patients exposed to 5-flourouracil and methotrexate, 32 were administered folinic acid as adjuvant therapy. Of the 36 patients exposed to either cyclophosphamide or ifosfamide, seven required rescue treatment with mesna. Additionally all the patients received a course of anti-histaminic and corticosteroid in pre-medication.

**Conclusion:**

Most common ADRs reported were alopecia, constipation, diarrhea, anorexia and neutropenia. Use of prophylactic and rescue medication reduced ADRs.

**014**

**Study of drug utilization pattern and their ADR (adverse drug reaction) monitoring in patients of viral hepatitis attending hepatology OPD of a tertiary care hospital**

Das P, Mohanty M, Patra UC, Mohapatra S, Patnaik KP

**SCB Medical College and Hospital, Cuttack, Orissa, India.**

Viral hepatitis is an infective disease affecting the liver predominatly and is caused by one of five viral agents : hepatitis A (HAV), B (HBV), C (HCV), D(HDV) and E (HEV). Along with specific antiviral drugs, various nonspecific drugs i.e. UDCA, Sylimarine etc. and various drugs used in traditional medicines are also used in viral hepatitis. So in this scenario, it has become essential to study the frequency of use of various medications including antiviral drugs in patents of viral hepatitis and to evaluate their relative cost of therapy, effectiveness and ADR. This study was done in Department of Pharmacology, in collaboration with Department of Hepatology, S.C.B, Medical College and Hospital, Cuttack, Orissa. Out of all patients attending Hepatology OPD with hepatic disorders, patients diagnosed as viral hepatitis were included. Patients attending with other liver disorders and diseases like carcinoma pancreas presenting with jaundice, gastrointestinal carcinoma, tubercular ascites etc. were excluded. The data were recorded in a predesigned proforma. Infectionwith HAV was more common. No specific antiviral therapy was given to patients with HCV infection, patients with HAV infection and patients with HEV infections were mainly treated with UDCA, Sylimarine, whereas patients with HBV infection were treated with antiviral drugs like entecavir, lamivudine and adefovir. Besides these, proton pump inhibitors, antiemtics and multivitamins were prescribed to almost all patients. In a single case of HBV infection, patient treated with lamivudine developed maculo papillary rash. This study will provide information regarding disease burden, prognosis, drug utilization pattern with their adverse effects alongwith cost-effective analysis of various drugs used in viral hepatitis in a tertiary care hospital in Eastern Orissa.

**015**

**Prescribing practices in neurology out-patient department in a tertiary care teaching hospital of northern India**

Singh J, Badyal DK

**Christian Medical College and Hospital, Ludhiana-141008, India.**

The principal aim of drug utilization research is to facilitate the rational use of drugs in population. Drugs used for CNS disorders are widely prescribed however the data on their utilization is limited in Indian studies. Three hundred (300) prescriptions of the out-patient department in Neurology OPD were analyzed. The data collected was used to evaluate the WHO drug use indicators: Average number of drugs per prescription, the percentage of drugs prescribed by generic names and percentage of drugs prescribed from essential drug list. The ATC / DDD methodology was used for the purpose of standardization. A total of 300 prescriptions containing 913 drugs were analyzed. Three drugs were prescribed on an average per prescription. Twenty percent of drugs were prescribed from essential drug list and 19.30% prescriptions were found to be incomplete. Only 12.8% of drugs were prescribed by generic names. One third (34%) prescriptions were for seizure disorders; out of which 29.9% (1/3^rd^) contained a combination of two or more anti epileptics. The five most common anti epileptic used were phenytoin (17.97%), valproate (14.04%), lamotrigine(11.79%), carbamazepine (11.79%) and divalproex(10.67%).Other than seizure disorder, prescriptions were for migraine (21.6%), cerebrovascular accidents (17%), neuropathies (8.6%), anxiety disorders(3.3%), parkinsonism (3%) and neurodegenerative disorders(1.6%). Our findings indicate a need for improvement in prescribing patterns especially in the area of generic names, essential drugs and writing complete prescriptions.

**016**

**A study of prescribing pattern in a tertiary care hospital**

Kumar S, Keshri US Pd, Gangopadhyaya T, Sharma J

**Rajendra Institute Of Medical Sciences, Ranchi, Jharkhand, India.**

**Objective:**

To evaluate and recommend modifications to achieve rational prescription.

**Materials and Methods:**

100 prescriptions from RIMS, Ranchi pharmacy center were collected where prescription comes from various departments and it was analyzed. Emphasis was given on core health care indicators particularly patient care and prescribing indicators.

**Results:**

The core indicators are: 1. Antibiotic percentage in a prescription 32; 2.Generic drug percentage in the prescription-81; 3.Average no. of drug per prescription-2.5; 4.Prescription percentage with dosage unclear-8 and 5.prescription percentage with unclear duration-6.

**Conclusion:**

Various factors influences the prescribing behaviors although drugs prescribed were mostly from national essential medicine list and were prescribed in generic names. There is need to fill all the information that is the part of a prescription.

**017**

**A comparative study of clinical efficacy and tolerability of second generation (cetirizine) and third generation (levocetirizine) antihistaminics in seasonal allergic rhinitis**

Rani S, Gupta MC, Verma P^1^, Singh D^1^, Walia R^1^

**Pt. B.D. Sharma Postgraduate Institute of Medical Sciences, Rohtak and Govt. Medical College, Patiala^1^, India.**

**Introduction:**

Newer antihistaminics are coming up for clinical uses with modifications in the chemical structure of the antihistaminics already in use e.g. cetirizine (racemate mixture) and levocetirizine (R-enantiomer). So a comparative study of clinical efficacy and tolerability of levocetirizine over cetirizine in seasonal allergic rhinitis was carried out.

**Methods:**

Sixty patients of seasonal allergic rhinitis were enrolled in an open, randomized and parallel group study as per predefined inclusion and exclusion criteria. The protocol was approved by the Institutional Ethical Committee and written informed consent was taken from all patients. Patients were randomly divided into 2 groups of 30 each. Group A received cetirizine 10mg once daily orally and Group B levocetirizine 5 mg daily for 4 weeks. Baseline total symptom complex score and nasal congestion score were noted prior to cetirizine and levocetirizine therapy and then weekly up to 4 weeks. Efficacy of treatment in study groups was measured according to total rhinitis symptom score and nasal congestion score whereas tolerability of the treatment regimen compared according to the observations of side effects. All observations thus made were statistically analysed.

**Result:**

Total symptom complex score was improved by 99% in group A and 98.3% in group B which was almost similar. The improvement in the Nasal Congestion score was significantly more (95.8% in group B as compared to 57.7% in group A) Side effects were comparable is except sedation and fatigue which were more in group B.

**Conclusions:**

Both cetirizine and levocetirizine were equally effective in controlling the seasonal allergic rhinitis but levocetirizine definitely scored over cetirizine as far as symptoms of nasal congestion were concerned and also has cause less side effects like drowsiness, fatigue.? Levocetrizine may be better option for the treatment of seasonal allergic rhinitis both in terms of efficacy and safety.

**018**

**Evaluation of quality of life in psoriasis patients**

Sarala N, Bhuvana K, Syed Yousuf Ali, Singh G

**Sri Devaraj Urs Medical College, Kolar, Karnataka, India.**

**Introduction:**

Psoriasis has profound impact on psychological and social aspects of an individual because of its visibility. This study was undertaken to assess quality of life (QOL) and clinical severity in psoriasis patients.

**Materials and Methods:**

This study was conducted by departments of Pharmacology and Dermatology at RLJ Hospital and Research centre attached to Sri Devaraj Urs Medical College, Kolar. Fifty patients of either sex with psoriasis were interviewed with questionnaire of 15 questions relating to how much psoriasis affected their lives. Patients were analyzed for age, sex, duration of disease associated disease, family history, questionnaire scoring and clinical severity of disease using psoriasis area severity index(PASI).

**Results:**

Among fifty patients 35 were males, 15 females with mean age of 41±13 yrs and 32±16 yrs respectively. 30 rural and 20 semiurban. 6 of them had one of the family member suffering from psoriasis. The mean duration of illness was 5±6 years. Areas affected were scalp(20), generalized(10), lowerlimbs(8), palms and soles(5), upperlimbs(5), trunk(2).Mean questionnaire scoring in males 11±7and females10±7. Mean PASI was 4.5±4.5 in males and 3.6±3.4 in females.

**Conclusion:**

We observed that psoriasis occurred at an early age in females than males. Duration of disease did not have any impact on QOL and clinical severity. Patients with positive family history had higher scores of QOL and PASI. Scores were higher in males could be because 8 out of 10 had generalized psoriasis. The relationship between QOL and PASI was insignificant could be due to milder disease which did not interfere with their life style

**019**

**Effects of oral montelukast as an add-on treatment in patients with moderately severe and stable chronic obstructive pulmonary disease**

Gupta MC, Chaudhary D

**Pt. B D Sharma Postgraduate Institute of Medical Sciences, Rohtak – 124001 (Haryana), India.**

**Introduction and Objectives:**

COPD is a serious illness that affects over 5% of the adult population for which morbidity and mortality is still on the increase. COPD is characterized by a progressive development of airflow limitation which is not fully reversible. Leukotrienes are known to play an important role in the pathogenesis of COPD. This study was carried out to evaluate the efficacy of Montelukast, a leukotriene antagonist as an add-on treatment in moderately severe COPD.

**Methods:**

A prospective, randomized, comparative, double blind, placebo controlled clinical study was carried out in 60 patients who were divided into two groups of 30 patients each to receive two different treatments. They had chronic airflow impairment defined as FEV_1_/FVC <70% and FEV_1_ <80% of the predicted value. Group I received standard treatment which comprised of tiotropium bromide (2 puffs of 9 *µ*g each) and formoterol (2 puffs of 12 *µ*g each)along with 10 mg oral montelukast while Group II received the standard treatment with a placebo. Primary parameters for efficacy were pulmonary function tests i.e. forced vital capacity (FVC), forced expiratory volume in first second (FEV1) ratio of FEV1/FVC and peak expiratory flow rate (PEFR). The secondary parameters were Borg dyspnoea score, β_2_ agonist sparing effect and sputum neutrophil count. The clinical assessment was made before drug administration and at 3 and 6 week post drug administration.

**Results:**

Montelukast caused a significant improvement in FVC, FEV_1_, FEV_1_/FVC and PEFR indicating an improvement in the lung functions. It caused a significant decline in the dyspnoea score and sputum neutrophil count. Montelukast also exhibited a β_2_ agonist sparing effect.

**Conclusions:**

Montelukast, a leukotriene antagonist may be a very useful drug in providing an effective add-on option for better management of the patients with COPD where the choice of drugs is often very limited.

**020**

**Pharmacogenomic, a concept for modern clinic**

Soni S, Bhardwaj S, Dholpuria R, Baniwal P, Thacker S

**Seth G. L. Bihani S. D. college of technical Euducation Pharmaceutical Science and Drug Research, Sriganganagar India.**

Today the experimental pharmacology has tremendously drifted from conventional to molecular and biochemical aspects. One may say pharmacologist followed the genetics to understand the variations in the individual drug response. Pharmacoginomics is an extension of pharmacogenetics and deal with hereditary impacts upon drugs action. Pharmacogenetics began with the study of single gene difference between individuals. Current medicine is based on statistical likelihood and often fails. The incidence of serious or fatal drug reaction can be minimize by using Personalized medicine. We have known for ages that drug is not equally effective in individuals. Pharmacogenomics gave us valuable tools to decipher the individual variations in gene. Using genotyping it is possible to consider the consequence of inter individual variation and this information can be used to design new drugs and to individualize drug therapy with existing drugs. The concept of modern clinic is based on the test for specific gene that will guide the doctors to prescribe medicine for best effectiveness and fewer side effects. To reach the aim, we must learn about the genetic variants that may affect the drug action. The most frequently occurring genetic variants are single-nucleotide polymorphism (SNP). Analysis of individual genetics variants can determine by following steps

- Extraction of DNA from patient’s blood sample
- PCR amplification of gene
- Fragmentation and labeling of the PCR product
- Hybridization and staining on the chip
- Scanning the chip
- Data analysis

B. How to chip analyses the snips analyze in patient after data analysis
  1. Individual SNP profile
  2. SNP profile and Response to drug therapy
  3. SNP and drug interaction
  4. SNP and Adverse Drug reaction
    The AmpliChip CYP450 test is the first FDA approved pharmacogenetic test on December 24, 2004.

**021**

**Oxidative stress and anti-oxidant status in breast cancer patients**

M Nagulu^1^, K Nalini^3^, R Dharak^3^, V Uday^2^, Reddy YN^2^, D Rama Krishna^2^

**^1^Department of Pharmacy Practice, Vagdevi College of Pharmacy, Warangal; ^2^Department of Pharmacology and Clinical Pharmacy, University College of Pharmaceutical Sciences, Kakatiya University, Warangal, Andhra Pradesh; ^3^Kakatiya medical College, Warangal, India.**

Breast cancer (BC) is a global public health problem as it is the third most common cancer leading to the death of women worldwide. Reactive oxygen species (ROS) damage DNA, but the role of ROS in breast carcinoma may not be limited to the mutagenic activity that drives carcinoma initiation and progression. Carcinoma cells *in vitro* and *in vivo* are frequently under persistent oxidative stress. Many references revealed that the low levels of antioxidants induce the generation of free radicals leading to DNA damage and further mutations. In the present study, an attempt has been made to evaluate the levels of serum Glutathione [GSH] and Total anti-oxidant status [TAS]. Total 65 subjects were selected for study. Out of 65 subjects 20 were normal healthy volunteers (control group) and 45 subjects were patients who are untreated with BC. The female patients within the age group of 25-65 years were selected. They were clinically and histopathologically diagnosed for BC. 20 healthy female subjects from the same economic status, having no history of smoking, alcoholism, any type of carcinoma etc were treated as controls. Significantly decreased values of GSH and TAS were observed (*P*<0.001) in untreated BC patients as compared with healthy controls. After therapy there was significant increase in the GSH and TAS as compared with untreated patients (*P*<0.001) From our findings it can be concluded that the oxidative stress is induced among BC patients, which in turn increases the risk of BC.

****

**022**

***In vitro* absorption studies of mucoadhesive tablets of acyclovir**

Dias RJ, Sakhare SS, Mali KK

**Department of Biopharmaceutics, Satara College of Pharmacy, Satara, Maharashtra, India.**

**Introduction:**

Acyclovir, an antiviral agent has less oral bioavailability, short plasma half-life and less presystemic metabolism. To increase the bioavailability of acyclovir, mucoadhesive drug delivery system was selected in which the dosage form is retained in the stomach so that it can be released for an extended period of time. The purpose of this study was to improve the absorption of acyclovir using sodium lauryl sulfate as permeation enhancer.

**Methods:**

Mucoadhesive tablets of acyclovir were prepared by direct compression method using polymers carbopol 934P and hydroxypropyl methylcellulose K100M. Dibasic calcium phosphate was used as pore forming agent and directly compressible lactose as a diluent. The chicken’s small intestine was used to study the permeation of mucoadhesive tablets of acyclovir. Dissolution-absorption studies were conducted on marketed and mucoadhesive tablets of acyclovir using varying concentration of sodium lauryl sulfate (SLS) as a permeation enhancer.

**Results:**

The results showed that marketed tablets of acyclovir had less permeability coefficient (0.778×10^-9^ cm/sec) as compared to mucoadhesive tablets with varying concentrations of SLS. The permeability increased with increasing concentration of SLS and permeability coefficient for mucoadhesive tablets with 4% SLS was found to be highest (5.231×10^-9^ cm/sec).

**Conclusion:**

Amongst the varying concentrations of SLS used, 4% of SLS in the dissolution medium of mucoadhesive tablet of acyclovir showed highest increase in permeation of acyclovir thereby increasing the bioavailability of acyclovir.

**023**

**A comparative study of tramadol and diclofenac (modified release preparations) in patient with burns**

Bhagat S, Kaushal S, Chopra SC, Uppal S

**Dayanand Medical College and Hospital, Ludhiana, India.**

Burns are amongst most severe forms of trauma and cause intense and prolonged types of acute pain, made worse by change of dressings, debridement procedures and infections. Undertreated pain can result in non-compliance with hospital treatment (dressings, diet, etc) and increased risk of post-traumatic stress disorder. Analgesia in burns could be achieved by pharmacological and non-pharmacological methods including opioids, nonopioids and adjuvant drugs. Patients fulfilling all the inclusion (20-60% Flame burns, age 15-60 years, presentation within 48 hours of burns) and none of exclusion criteria (H/o idiosyncrasy/ hypersensitivity to any of study drug, CRF, Chronic hepatitis, Bronchial asthma, Bleeding disorder, Acid peptic disease, Drug addicts) were randomly assigned to either of two treatment groups, with modified release preparations of Tramadol (T) and Diclofenac (D) after informed consent. Patients were assessed twice daily for two weeks for pain severity, character, localization, adverse effects and tolerability. Injection morphine 4 mg I/V was administered as an escape treatment. Statistical significant reduction in pain score was achieved in both groups as compared to baseline VAS scores, (*P*= 3.18X10^-11^ (T), *P*= 9.71X10^-10^ (D) group), T produced more reduction in pain scores as compared to D (*P*= 0.001887) but without any significant difference in adverse effect profile. Tramadol produced significant reduction in pain scores as compared to Diclofenac without any increase in adverse effects in patients with burns.

**024**

**Drug utilization pattern in cases of hypertension in a tertiary care hospital**

Badwaik RG, Giri RR, Patil AB, Phadke AG, Jha RK, Patel SS

**Jawaharlal Nehru medical college, Sawangi, Wardha, India.**

**Introduction:**

The present drug utilization study was undertaken to establish the prescribing pattern of antihypertensive drugs in a tertiary care hospital.

**Materials and Methods:**

Prescription files from the period of July 2007 to December 2007 were selected from the Medical Record Department of Acharya Vinoba Bhave Rural Hospital Sawangi (M), Wardha. Total 270 prescriptions having diagnosis of hypertension were analyzed for age, gender, diagnosis, drugs prescribed and the data was collected.

**Results:**

Among 270 prescriptions analyzed, monotherapy with a single antihypertensive agent has been used in 144 (53.3%) patients, whereas combination therapy with two or more drugs has been used in 126 (46.7%) patients. In monotherapy, Calcium channel blockers were given to 46 (31.94%) patients, Beta blockers 41 (28.47%) patients, ACE inhibitors 36 (25%) patients and diuretics 21 (14.58%) patients. Among monotherapy Calcium channel blockers were the most commonly prescribed drugs, whereas diuretics were the least commonly prescribed drugs. In combination therapy, a combination consisting of two drugs was given to 107 (84.92%) patients whereas combination consisting of three drugs was given to 19 (15.08%) patients. A two drug combination consisting of Calcium channel blocker and beta blocker was given to majority of patients.

**Conclusion:**

Our study shows that there is more preference for the monotherapy but underutilization of diuretics in the present prescribing pattern and As per JNC 7 guidelines, there is considerable scope of improvement by increasing there utilization.

**025**

**Randomized double blind placebo controlled trial to demonstrate the role of zinc in recurrent acute lower respiratory infections**

Ahmad F, Shah UH, Malik MA, Alam S

**J.N. Medical College and Hospital, Aligarh Muslim University, Aligarh.**

**Introduction:**

Acute lower respiratory infections (ALRI) account for about 1/3 of childhood deaths. These are associated with malnutrition and reduced immunological incompetence which may be attributable to Zinc deficiencies. So this study was conducted to observe the effect of Zn supplementation on the morbidity pattern of children with recurrent ALRI.

**Materials and Methods:**

Study was conducted April 2006 to September 2007 in Paediatrics OPD. Children aged 6 months to 5 years with recurrent ALRI as per WHO definition were enrolled. Children with known causes of recurrent respiratory symptoms, any other active illness, WAZ score or HAZ score less than -2 and having received Zinc in past 3 months were excluded. After taking informed consent, 52 cases of ALRI were included which were randomized into two groups, 26 each to zinc group and placebo group. 52 age and sex matched children were taken as controls. Serum Zinc was estimated and morbidity data were collected fortnightly.

**Results:**

Out of 216 children who presented with repeated episodes of respiratory illness 52 were included. Mean serum zinc levels increased from 46.6± 18.4 to 75.4 ±21.7 microgram/ dl in zinc group, whereas it decreased from 50.7±14.2 to 42.8± 13.7 microgram/dl in placebo group. The mean episode of ALRI in zinc groups (1.04 ± 0.75) was lower as compared to placebo group (1.458 ± 0.78). There was statistically significant difference in episodes of severe ALRIs between the two groups (0.22 ±.421 vs 0.54 ± 0.51), *P* value 0.02. The mean ALRI free days and morbidity free days were also more in zinc supplemented group.

**Conclusion:**

Our study shows zinc supplementation decreases respiratory morbidity and incidence of severe acute lower respiratory infections.

**026**

**Clinical research regulations in India: A metaanalysis and rational in global prospective**

Vetal Y^1^, Harle UN ^2^, Divekar GS3, Patil M ^1^

**^1^AISSMS College of Pharmacy, Pune, ^2^University Department of Pharmaceutical Sciences, Nagpur, ^3^Sun Pharmaceutical Industries Ltd, Mumbai, India.**

Schematic review and meta-analysis was performed to determine the clinical research practices followed in India comparing other countries. Meta-analysis of episode studies eligible for this systematic review included those in which mortality data were presented in epidemiology revealing malpractices of clinical research. After independent quality assessment and data extraction, data were pooled for meta-analysis. The placebo, randomized and blinded clinical trials design were evaluated to be showing high risk of mortality. The meta-analysis indicated that current clinical practices in India is associated with diminished overall quality and accountability and further increased the risk with current clinical research practices. The mortality in clinical trials was correlated with significantly less audit and inspection from regulatory authority. Predominant difference in regulations were found to be weak clinical trial review process as per the functions depicted to IEC; IND and NDA intermittent review process is not precisely defined, specific guidelines in special studies are obscured, colour and additives are not covered, delegation of liabilities to state authorities are marginal, frail Pharmacovigilance publication, etc. US-FDA have reported the deficiencies in Good Clinical Practices as Inadequate Consent form 46%, Protocol violation 31%, Inadequate/inaccurate records 26%, Poor drug accountability 19%, Failure to inform IRB 7%, Inadequate adverse event reporting 4%, IRB approval not obtained 2%, Failure to obtain patient consent 1%. It may conclude that rational approach using meta-analysis may be employed to clinical research design, conduct and regulation towards reducing the risk in clinical trials.

**027**

**A prospective study on the effects of diclofenac sodium and etoricoxib in the treatment of osteoarthritis**

Jain A, Jain IP, Singh SP, Singh S

**G.S.V.M. Medical College, Kanpur, Uttar Pradesh, India.**

**Objective:**

To compare the gastrointestinal tolerability, safety, and efficacy of diclofenac and etoricoxib in patients with osteoarthritis.

**Materials and Methods:**

A prospective study was conducted in O.P.D. of orthopaedics department for two months at LLR hospital Kanpur. A randomized, double-blind control trial was done on 644 patients over 40 years with uncomplicated osteoarthritis knee. Patients were divided into 3 groups, Group I (n=215) received diclofenac sodium(100 mg OD SR), Group II received (n=216) etoricoxib 90 mg OD and Group III(n=213)received placebo.

**Inclusion Criteria:**

Males or females over 40years of age with osteoarthritis knee having symptoms(joint pain, morning stiffness <30 min, restricted mobility) for at least 3 months with radiological evidence.

**Exclusion criteria:**

Major cardiac disease, severe hepatic disease, bronchial asthma, active GI bleeding, allergy to NSAIDS, steroids, warfarin, lithium. The primary endpoint was the discontinuations due to GI adverse effect.

**Results:**

644 patients were studied. 248(38.5%)were males, 396(61.5%) were females. Male:female ratio was 1:1.6.Patients belonging to age group 40-50years-106(16.5%), 50-60years: 247(38.4%), over 60 years: 291(45.1%). Therapy was completed by 153(71%) patients on diclofenac, 187(86.3%)patients on etoricoxib, 133(62.4%)patients on placebo. Discontinuation rate due to GI Adverse effects was significantly lower with etoricoxib than diclofenac. Discontinuation rate due to hypertension-related Adverse effects was higher with etoricoxib than diclofenac(2.2%vs0.6%). Etoricoxib and diclofenac showed similar efficacy.

**Conclusion:**

This study shows that number of osteoarthritic females were1.6 times than that of males. Most of patients belong to elderly age group(>60 years). The commonest cause of discontinuation of therapy was unacceptable adverse effects.Etoricoxib was associated with better GI tolerability, better clinical efficacy compared to diclofenac.

**028**

**Evaluation of plant isoflavone effect on platelet aggregability and RBC antioxidant parmamters in oophorectomised women: A randomized double-blind placebo controlled clinical trial**

Hota D, Mittal N, Dutta P, Suri V, Ahluwalia J, Chakrabarti A

**Postgraduate Instutute of Medical Education and Research, Chandigarh, India.**

Phytoestrogens by virtue of their potential to act as selective estrogen response modifiers (SERMs) and cardioprotective effects have received attention as alternatives to hormone replacement therapy for post menopausal women. Inhibition of platelet aggregation and antioxidant action are among the number of mechanisms postulated to be responsible for their cardioprotective effects. This randomized, double blind, placebocontrolled trial evaluated the effect of soy isoflavones (75 mg/day for 12 weeks) on ADP and epinephrine induced platelet aggregation and RBC antioxidant parameters (lipid peroxidation, superoxide dismutase, catalase, glutathione peroxidase) in 34 women who had undergone bilateral oophorectomy. The outcomes were assessed at baseline and 12 weeks after randomization to either group. The two study groups were comparable in terms of demographic, clinical characteristics, platelet aggregation and RBC antioxidant parameters at baseline. There was no significant alteration in ADP and epinephrine induced platelet aggregation and RBC antioxidant parameters (lipid peroxidation, superoxide dismutase, catalase, glutathione peroxidase) in either of the two groups at 12 week compared to the baseline. Mean change in the various parameters at 12 week from baseline also did not show significant difference between two groups. Isoflavones (75 mg of isoflavones daily for 3 months) did not produce any significant alteration in ADP and epinephrine induced platelet aggregation and RBC antioxidant parameters suugesting their safety. However, future studies are needed to provide a conclusive proof regarding the use of isoflavones in clinical practice.

**029**

**Prescription pattern of nonspecific upper respiratory tract infection in ent outpatient department of tertiary care hospital**

Jain V, Jain IP, Singh SP, Singh S, Saeed S

**G.S.V.M Medical College, Kanpur, India.**

**Objective:**

To analyze the prescription pattern of nonspecific upper respiratory tract infection in ENT outpatient department of LLR hospital Kanpur.

**Materials and Methods:**

A prospective study of prescription scripts was done from 1^st^ July 08 to 30^th^ July 08. Prescription related to nonspecific respiratory tract infection were collected and scrutinized. Study include classification of drug, number of drugs per prescription, age and sex distribution and advice related to upper respiratory tract infection.

**Results:**

484 prescription scripts were analyzed. The age of patient ranged from 15-65 years. Age group distribution was <20yrs -15.3%, 20- 40yrs-47%, 40-60yrs-29.3%, > 60yrs-7.4%.There were 55% male and 45% female patients. The overall mean of total number of drug per prescription was 3.2.Frequency of prescribing antimicrobial is 100% out of which 85.1% is cephalosporins, 8% macrolide, 4.95%quinolones, 1.85% others. Frequency of antihistaminic is 92%, analgesic 57%, vitamins B complex 66%, antacids 23%, gargles 9%, advice of rest, gargles, increase fluid intake found only in 13% of 4prescription.

**Conclusion:**

This study provides a baseline data for monitoring future prescribing trends. Theory states that most of the nonspecific upper respiratory tract infection caused by viruses so it is irrational to use antimicrobials in every case and advice related to rest, gargles, increase fluid intake which are mandatory in upper respiratory tract infection is missing from most of prescription so there is need of regular auditing and discussion with prescribing physician for improving prescribing habit.

**030**

**Analysis of fixed dose combinations of vitamin A, folic acid and iron for use in pregnancy**

Gupta Rachna

**Dept of Pharmacology, Indraprastha Dental College and Hospital, Ghaziabaad, U.P. India.**

**Background:**

A number of preparations containing Vitamin A, folic acid and iron in varying amounts are available in the market. The variation of amount of components can result in adequate intake of some while inadequate/excess intake of others. This becomes crucial in pregnancy when deficiency of any of these can result in a number of complications.

**Materials and Methods:**

All fixed dose preparations of iron with folic acid and / or vitamin- A that are included in May- June 2008 issue of IDR were analyzed. Dose needed to meet iron needs in a pregnant female was calculated for each preparation. At the same dose intake of other components- Vitamin A and folic acid for each preparation was also calculated.

**Results:**

A total of 443 preparations were analyzed. Only 77 % provided appropriate amount of Vitamin A and/or folic acid in pregnancy.

**Conclusion:**

Special attention needs to be paid regarding choice of fixed dose preparations of Vitamin A, folic acid and iron in pregnancy.

**031**

**A drug utilization study of antimicrobial agents in the intensive care units in a medical college hospital of North India**

Gupta A, Mahajan B, Garg S, Kaushal S, Chopra SC

**Dayanand Medical College and Hospital, Ludhiana, India.**

The APACHE (Acute Physiology and Chronic Health Evaluation) is prognostic scoring system for classifying patients in ICU on the basis of physiological scores and current health status. The chances of survival increase with a decrease in the score. AMAs play a major role in management of such illnesses and can dramatically improve patient outcome. This study was undertaken to study AMA use and patient outcome in each category of APACHE II. Data was collected from 49 patients admitted in Surgery and Neurosurgery ICUs after IEC approval. APACHE II score calculation was done at 24 hours of ICU-admission. The use of AMAs was studied in terms of average number of AMAs used per patient in each category of APACHE score; most commonly prescribed AMA in each category and its route of administration. The patients who went LAMA were excluded from the outcome study. The highest use of AMAs was 4.3 in APACHE score group >25; and least was 3.1 per patient in 16-20 score group, overall average being 3.61 per patient. The most commonly used AMA group was cephalosporins and route of administration was intravenous. There was a significant difference in the outcome of patients of both the ICUs amongst the patients shifted to the wards and patients who died (*P* = 0.03). AMA use policy is in place. There is no difference in the quality of care among the patients in either of the ICUs and sub categories of APACHE scoring.

**032**

**Response of long term aspirin administration in patients of ischemic heart disease**

Kaura R, Laller KS, Singh J, Sen R

**Department of Pharmacology, Cardiology, Pathology P.G.I.M.S. Rohtak, India.**

**Objective:**

To study the effect of long term aspirin administration on platelet aggregation and adhesiveness in patients with ischemic heart disease.

**Materials and Methods:**

Thirty male patients aged (40-75yrs) who attended cardiology OPD with evidence of stable angina and thirty age matched healthy volunteers were enrolled for the study. Group I- Control group; Group II- Aspirin treated. Platelet aggregation and adhesive indices in control, aspirin (150mg, p.o., daily for 12 months) treated patients were estimated by O’Brien and Salzman’s method. Blood samples for platelet aggregation and adhesiveness were collected in separate vials containing sodium citrate and EDTA respectively. Platelet rich plasma (PRP) was obtained by centrifuging blood at 150-200 G for15 minutes. Platelet aggregation was induced by adding 20 *µ*g of ADP. Platelet aggregation induced alterations in optical density were measured with photoelectric colorimeter.

**Results:**

Platelet aggregation time was significantly (*P* <0.01) increased in patients with angina treated with aspirin (150mg, p.o., daily for 12 months). Platelet adhesive index was found to be significantly reduced in these patients after aspirin treatment. Responsiveness of aspirin after 12 months of treatment was reduced but not upto a significant level.

**Conclusion:**

In patients with stable angina, aspirin resistance was not observed after 12 months of treatment.

**033**

**Antioxidant studies on ethanolic extract on *Commiphora* sp leaves**

V Sudarshana Deepa, P Suresh Kumar, P Selvamani, S Latha, S Srinivasan

**Anna University Tiruchirappalli, Tiruchirappalli -620024, Tamil nadu, India.**

This investigation elucidated the role of phytochemical constituents and *in vitro* free radicals scavenging activity for nitric oxide, total reducing power, superoxide, lipid peroxidation and DPPH in the ethanolic extract of *Commiphora* sp leaves–*Commiphora caudata* and *Commiphora var pubescens*. The IC_50_ values of both the species were comparable to standard drugs- Quercitin (nitric oxide), Vitamin C (superoxide), Vitamin E (lipid peroxidation), Vitamin C (DPPH).The results were analyzed statistically by regression analysis. In all the invitro assays the ethanolic extracts of the leaves showed its ability to scavenge free radicals in a dose dependent manner. The results revealed that *Commiphora caudata* has potent antioxidant activity better than *Commiphora var pubescens*. Further investigation on the isolation, identification of antioxidant components in these plants may lead to chemical entities with the potential for clinical use and evaluation of *in vivo* antioxidant activity.

**034**

**A study of habit of computer and internet usage among medical professionals**

Gupta MC, Verma S, Singal R, Jindal P

**Pt. B.D.Sharma, Post Graduate Institute of Medical Sciences, Rohtak, India.**

**Objective:**

Internet has become the world’s biggest library, where retrieval of scientific information can be done within minutes and it has revolutionised the medical practice with the increasing use of telemedicine and evidence based medicine. The present study was conducted to assess the habit of computer and internet usage amongst medical professionals.

**Methods:**

A detailed questionnaire was designed to assess the trend of computer and internet use amongst the medical professionals in PGIMS, Rohtak. A total of 200 medical professionals including consultants(C), senior residents (SR) and post graduates (PG) were included in the study. The responses as per the questionnaire were recorded and analysed.

**Results:**

Out of the 200 subjects involved in the study, 189 (94.5%) were using computers regularly while 11 (5.5%) had no exposure to the computers at all. Most users (C-85.9%, SR- 78.4%, PG-87.6%) were found to independently use the computers while some users had some form of assistance (C-31.5%, SR- 25.4%, PG-25.9%). The computer usage was most common at home (78.5%), followed by cyber cafe (33.7%) and the college (30.2%). The purpose of using computers was for seeking general information (74.2%), research work (58.9%) and entertainment (46.1%). The internet use related to research work was 71.9% in consultants, 29.4% in senior residents and 75.4% in post graduates. The frequency of the internet usage was found to be 2-3 times /week. The reasons for using internet for seeking information were a quick access (51.7%) to the latest information and easy to use (45.8%). Commonly used websites were google, pubmed and yahoo. The percentage of users ensuring quality assurance was found to be very low (C-33.3%, SR-25.4%, PG-24.6%). Most of the users (C-77.2%, SR-64.7%, PG-76.5%) were of the view that computer education is a must in teaching institutions. A larger number of users (C-54.4%, SR-64.8%, PG-66.7%), were unaware of computer assisted teaching.

**Conclusion:**

The use of computers and internet for seeking variety of information though has become popular yet a significant number of medical professionals are still not using computers. The medical institutions need to promote the use of computers and internet amongst the faculty and residents. The concept of computer assisted teaching needs to be introduced not only for the teaching faculty but also for the post graduate and undergraduate students.

**035**

**Acute effects of tiotropium alone and in combination with formoterol in patients with COPD: comparison of three regimens**

Imran M, Kotwani A, Chhabra SK, Vijayan VK

**Vallabh Bhai Patel Chest Institute, University of Delhi, Delhi – 110007, India.**

**Introduction:**

The data on combination of long-acting β_2_ agonists and anticholinergics in the treatment of COPD are lacking. This study compared the bronchodilator effects of tiotropium, formoterol and both combined in chronic obstructive pulmonary disease (COPD).

**Methods:**

A total of 60 COPD patients (mean forced expiratory volume in 1 second FEV1 ≥ 50% predicted) participated in this randomized, double-blind, placebo-controlled, active-drug controlled study and received tiotropium 18 *µ*g in the morning once, tiotropium 18 *µ*g and formoterol 12 *µ*g combination once in the morning and combination of the two drugs in the morning and formoterol 12 *µ*g in the evening. The end-points were 24 hrs serial spirometry (FEV1 and FVC) and Borg.s scales readings.

**Results:**

Addition of formoterol enhanced the peak effect in FEV_1_, FVC and this enhanced effect remains till the next dose (24 hours). Second dose of formoterol improved the lung functions (FEV_1_ and FVC) in the night and the values were highest in the next day morning (24 hours). Magnitude of difference at 24 hours in FVC (percentage) was statistically more with addition of second dose of formoterol in the evening but not in FEV_1_ (L) and FVC (L).

**Discussion and Conclusion:**

Addition of formoterol to tiotropium has shown improvement in lung function in COPD. In mild COPD, addition of second dose of formoterol in the evening to the combination (tiotropium and formoterol) showed superior effects. In moderate COPD patients tiotropium alone is as good as combination of the two given once in the morning.

**036**

**Comparision of efficacy and safety of sibutramine and orlistat in obese hyperlipidemic patients**

Krishan Pawan^1^, Kumar Raghuvansh^2^, Pawar Prafulla^1^

**^1^Punjabi University, Patiala, ^2^Government Medical College, Patiala, India.**

**Introduction:**

Obesity is a serious medical condition that like other chronic diseases requires treatment. Obesity is associated with dyslipidemia, diabetes, cardiovascular disease and hypertension. The present study has been designed to compare the efficacy and safety of sibutramine and orlistat in obese hyperlipidemic Patients.

**Materials and Methods:**

The study was carried out in 50 obese hyperlipidemic patients. The patients were randomized to receive either sibutramine (10mg) o.d or orlistat (120mg) t.i.d. for a period of 12 weeks. The study was undertaken after obtaining informed consent from the patients and the study was carried out in accordance with principles of declaration of Helsinki. For assessing efficacy, various anthropometric parameters viz; body weight, body mass index (BMI), waist circumference (WC), waist hip ratio (WHR), skinfold thickness and lipid profile, were measured at baseline and at the end of study. For evaluating safety, various hematological and biochemical parameters were assessed.

**Results:**

Both the drug treatments i.e. sibutramine and orlistat produced significant reduction in various anthropometric parameters i.e. body weight, BMI, WC, WHR, skinfold thickness and lipid profile. However as compared to each other, it was observed that sibutramine was more potent and efficacious in lowering body weight and BMI as compared to orlistat. However orlistat was found to lower lipid profle to greater extent than sibutramine.

**Conclusion:**

Sibutramine is a promising drug in the prevention and progression of obesity in obese patients and orlistat is beneficial in preventing obesity associated hyperlipidemia in obese hyperlipidemic Patients.

**037**

**A study of drug information seeking behaviour in medical professionals**

Gupta MC, Verma S, Jindal P, Singal R, Kaushal J

**Pt B D Sharma Post Graduate Institute of Medical Sciences, Rohtak (Haryana), India.**

**Objective:**

The present study was conducted to evaluate the drug information seeking behaviour amongst medical professionals.

**Methods:**

A cross sectional study was conducted at PGIMS, Rohtak and a detailed questionnaire was designed to assess the drug information seeking behaviour amongst 200 medical professionals.

**Results:**

Medical professionals are using variable sources for gathering information about drugs. Consultants (77%) were found to be more willing to regularly update their knowledge about drugs as compared to SR’s(65%) and PG’s (58%). The most common reason for seeking information was about new drugs for the consultants and senior residents(C-63%, SR-55%, PG-53%) while for post graduates it was recent advances in the drug treatments (C-49%, SR-47%, PG-65%). Other mediums for seeking drug information were found to be therapeutic guidelines (C-33%, SR-43%, PG-44%), adverse drug reactions(C-21%, SR-33%, PG-30%), indications and contraindications(C-32%, SR-47%, PG-46%), solving therapeutic problems and evidence based medicine (C-19%, SR-23%, PG- 30%), poisonings and overdoses(C-16%, SR-29%, PG-25%) and information on pricing and availability(C-14%, SR-25%, PG-16%). The preferred medium for updating drug related information in all the groups was books (C-67%, SR-63%, PG-70%) followed by the internet(C-49%, SR-55%, PG-64%) and journals(C-47%, SR-47%, PG-53%). Other mediums were information from peers, medical representatives and pharmacopoeias.

**Conclusions:**

The findings indicate that consultants were much more willing to pursue new information about the drugs as compared to their junior counterparts. Books followed by the internet were the most preferred sources for drug information. Considering high prevalence of irrational prescribing, the drug information seeking behaviour needs to be enhanced especially amongst the senior residents and post graduates who are not only the potential prescribers but also the future consultants.

**038**

**High density lipoprotein stimulates the activity of hepatic superoxide dismutase *in vitro***

Chander R^1^, Khanna AK^2^, Saxena JK2, Mahdi AA^3^, Mahdi F^1^

**^1^Era’s Lucknow Medical College, ^2^Cental Drug Research Institute, ^3^Chhatrapati Shahuji Maharaj Medical University, Lucknow, India.**

Most of the metabolic diseases are associated with imbalance between antioxidant-oxidant system and often caused decrease in High density lipoprotein (HDL), demunation of antioxidative enzymes and produce oxidative damage in body. We investigated stimulatory action of HDL on superoxide dismutase (SOD) activity *in vitro*. Rat plasma HDL was prepared by polyanionic precipitation method. Rat liver post mitochondrial supernatant was treated with ammonium sulphate and a Copper-Zinc superoxide dismutase (Cu-Zn SOD) rich fraction was prepared: The reaction mixture comprised of enzyme source (=180 *µ*g protein) NADH, Phenazine Methosulphate and Nitroblue Tetrazolium, in absence or presence of different concentrations of HDL (40-500 pmol) and was assayed for SOD activity on spectrophotometer at 560nm. In another set of experiment, superoxide anions (O_2_^-.^) were generated in two *in vitro* systems comprising of xanthine-xanthine oxidase as well as NADPH-resorscinol- MnCl2, in absence or presence of HDL (40-500 pmol) and assayed spectrophotometrically. Our data show that addition of HDL in the reaction mixture containing enzyme source, stimulated the activity of Cu-Zn SOD in concentration dependent manner, which was 176%, 300 pmol of this lipoprotein. HDL also inhibited the formation of O_2_ - as assessed by inhibition of xanthine oxidase activity (43%) and oxidation of NADPH (50%) at 120 and 210 pmol, concentrations respectively. Our finding suggests that stimulatory effect of HDL on rat liver Cu-Zn SOD may be due to antioxidant property of this lipoprotein. Furthermore, any disorder with HDL metabolism may directly affect the activities of antioxidant enzymes in the body.

**039**

**Antihypertensive efficacy of labetolol and nifedepine in patients of pregnancy induced hypertension along with preterm labor—a comparative study**

Singh R^1^, Singh SP^2^, Jain IP^3^, Singh S^4^, Agarwal P^5^

**GSVM Medical College, Kanpur, UP, India.**

**Objective:**

To study and compare the effects of Labetolol and Nifedepine on blood pressure (BP) and pregnancy outcome in patients of pregnancy induced hypertension, with preterm labor.

**Materials and Methods:**

Study was conducted in Obstetrics department of LLR hospital, Kanpur, for two months. 24 patients of pregnancy induced hypertension (PIH) presenting with labor pains at gestational age of 28-36 weeks were recruited for the study. Patients were randomized into two groups (n=12). Group1 receiving Labetolol 200mgBD and group2 Nifedepine 20mgOD orally daily. They were analyzed for reduction of systolic (SBP) and diastolic (DBP) blood pressure and for pregnancy outcome.

**Results:**

108 patients were admitted with labor pains, in which 46(42.6%) presented with PIH and only 24(22.2%) were between 28-36 weeks. In group1, 10(83.33%) showed reduction in SBP by 20±4 mmHg and DBP by 10±4mmHg while in group2, 11(91%) showed reduction in SBP by 26±2mmHg and DBP by 20±2mmHg. In group1, 10(83.33%) underwent preterm delivery and 2(16.66%) died of uncontrolled hypertension and in group2, 7(58.33%) were delivered premature, 1(8.3%) died of uncontrolled hypertension and in 4(33.3%) labor pains subsided.

**Conclusion:**

Clinically equivalent decrease in BP was observed with both drugs. Nifedepine reduces DBP more and early while Labetolol has better efficacy for gradual control of SBP andDBP. Nifedepine, being a tocolytic, subsides uterine contractions and extend the pregnancy towards term leading to better pregnancy outcome. So Nifedepine should be given in patients of high DBP and pregnancy not closed to term while Labetolol should be given in pregnancy closed to term as it is only antihypertensive, not tocolytic,

**040**

**Study of self-medication and drug abuse by medical students in a tertiary care teaching hospital**

Dutta SB, Keshari SS, Acharya SB

**SGRR Institute of Medical and Health Sciences, Dehradun, India.**

**Objective:**

To study self-medication and drug abuse done by medical students of a tertiary care reaching hospital in SGRR Institute of Medical and Health Sciences, Dehradun.

**Methods:**

A total number of 200 hundred students were included in the study. Prepared questionnaires were distributed to all these students and were collected after filling them and were analysed.

**Results:**

From the present study, it was found that most of the students were indulged in self medication for headache, cough and cold, diarrhoea, fever, abdominal pain, vomiting, insomnia and weakness. The most commonly used medication were NSAIDs, antimicrobial agents, cough syrup, spasmolytics, anxiolytics, appetizers, and multivitamins and minerals. Beside these some students were taking nicotine (chewable and smoking) and alcohol occasionally.

**Conclusion:**

Self-medication is harmful and may lead to life threatening adverse drug reactions. Indiscriminate use of NSAIDs may lead to peptic ulcer. Similarly indiscriminate and inappropriate use of antibiotics may lead to emergence of drug resistance. Anxiolytics may lead to drug dependence. Use of nicotine and alcohol in medical students may compromise their carrier and health in future.

**041**

**Pain relievers in acute renal colic**

Saluja T^1^, Sharma RA^2^, Sharma P^1^, G Mohan^1^, Kulshreshtha S^1^ Goyal J^1^

**^1^S.N Medical college, Agra, ^2^LLRM Medical College, Meerut, India.**

**Introduction:**

Acute renal colic is one of the most severe pain known to mankind and yet ideal analgesic remain unknown. We examined the relative benefits and disadvantages of fixed single bolus of intramuscular doses of NSAIDs and Opioids to determine the appropriateness of management.

**Methods:**

Randomised division of 130 pts of acute renal colic were done in 4 groups and were given single bolus of I/M injection of drug according to group alloted. Group1 (36) : Diclofenac, Group 2 (34): Piroxicam, Group 3 (32): Tramadol, Group 4 (28): Butorphanol and were evaluated for 1) Pt. rated pain score at 30 min. (VAS) (2) Failure to achieve complete pain relieve at 60 min. (3) Requirement of rescue analgesia between 30-60 min. (4) Side effects.

**Results:**

Both NSAIDs and opioids demonstrated a clinically important analgesic effects with a more than 50% reduction in pain score at 30 mint in 64.74% and 50% respectively. There was no significant difference between NSAIDs (69.11%) and opioids (62.90%) in the proportion of Pts. who achieved complete pain releive in a short time frame. But the rate of achieving complete pain relief is more with NSAIDs especially diclofenac (72.22%) Although both NSAIDs and opioids lead to clinically significant analgesic, a greater no. of Pts who received opioids (33.87%) required an additional dose of a rescue analgesic as compared to Pts receiving NSAIDs (13.23%) Adverse effects were generally higher in Pts receiving opioids (25.86%) as compared to NSAIDs (7.35%).

**Conclusion:**

While choosing a single bolus of analgesia, NSAIDs especially Diclofenac rather than opiods should be the choice.

**042**

**Study of antibiotic sensitivity pattern in urinary isolates**

Keshari SS, Ravikant, Dutta SB, Acharya SB

**SGRR Institute of Medical and Health Sciences, Dehardun, India.**

**Objective:**

To evaluate the spectrum of urinary isolates and their antibiotic sensitivity pattern in out-patients and indoor patients of Urinary Tract Infection (UTI).

**Materials and Methods:**

In a prospective study conducted at departments of pharmacology and medicine in SGRR Institute of Medical and Health Sciences, Dehradun. Uropathogenic strains of bacteria from out-patients and indoor patients were studied from August 2007 to August 2008 for their antibiotic susceptibility profiles.

**Results:**

Out of 280 suspected cases of UTI, significant higher degree of bacteriuria was detected in 88 cases. The commonest microorganism isolated was *Escherichia coli* followed by *Klebsiella* spp., *Proteus, Enterobacter, Citrobacter, Pseudomonas* spp. *Staphylococcus* spp., *Acinetobacter*. Antibiotic susceptibility pattern of these isolates revealed that aminoglycosides, nitrofurantoin, third generation cephalosporins, β-lactam β-lactamase inhibitors and fluoroquinolones were sensitive *in-vitro*. The cotrimoxazole, ampicillin, and 1^st^ and 2^nd^ generation cephalosporins were found to be resistant in bacterial isolates.

**Conclusion:**

Routine urine culture and sensitivity should be done in all the indoor patients before advocating treatment since there is significant variation in the antibiotic susceptibility pattern of urinary isolates in patients of UTI. This provides rational treatment, prevents emergence of drug resistance, and discourage clinicians to indiscriminate use of antibiotics.

**043**

**Comparative effect of captopril (ACE inhibitor) and losartan (AT_1_ antagonist) in ethanol induced gastric ulcer**

Das MK^1^, Sagar BPS^1^, Das S^2^, Ganti SS^3^, Das S^4^

**^1^IEC College of Pharmacy, Greater Noida, U.P., India; ^2^Noida Institute of Engineering and Technology, Greater Noida, U.P., India; ^3^ISF College of Pharmacy, Moga, Punjab, India; ^4^Delhi Institute of Pharmaceutical Sciences and Research, New Delhi, India.**

The mechanism behind ACE inhibitors (ACEI) is increasing PG synthesis, which causes a rapid increase in production of mucus. In contrast AT1 receptors antagonist inhibit PG level. In the same time these decrease TNF-alpha, ICAM-I and neutrophil infiltration in gastric mucosa. In present study the efficacy of Losartan in ulcer protection was estimated, which was again compared with Captopril. Rats were fasted for 24 hrs and were given 80% ethanol at a dose of 1 ml/ rat orally. The study groups received captopril (10 mg/kg, i.p.) and losartan (10 mg/kg, i.p.) 1 hr. before ethanol administration. Two hr. later, animals were sacrificed and ulcer score, ulcer index and mucus layer weight was determined. In the standard group (Ranitidine 50mg/kg), ulcer score and ulcer index was 1.33**±0.221 and 0.1026**±0.005 respectively, whereas in case of Captopril, it was 1.62**±0.206 and 0.1314**±0.003 respectively. In case of Losartan it was 1.66**±0.187 and 0.166**±0.0032 respectively. Mucus layer weights were 39.19**±0.72 mg and 30.03ns±0.81 mg in Captopril and Losartan groups respectively. Losartan decrease PG synthesis, but by decreasing TNF-alpha, ICAM-I and neutrophil infiltration in gastric mucosa it shows antiulcer activity. In case of Captopril it is by increasing PG synthesis. As Losartan does not increase PG synthesis, so it has no significant effect on mucus weight. In this present study it was observed that Captopril as well as Losartan showed ulcer preventive activity in ethanol induced gastric ulcer.

**044**

**A comparative study of amisulpride versus olanzapine in the treatment of schizophrenia**

Bhowmick S^1^, Hazra A^1^, Ghosh M^2^

**^1^Institute of Postgraduate Medical Education and Research, ^2^Bangur Institute of Neurosciences and Psychiatry, Kolkata, India.**

**Introduction:**

Atypical antipsychotics are increasingly drugs of first choice in schizophrenia. Amisulpride, a new atypical antipsychotic, has been reported to be effective for both positive and negative symptoms of schizophrenia in western countries but Indian experience is limited. We conducted a randomized controlled trial of amisulpride versus olanzapine in schizophrenia of at least 1 year duration.

**Methods:**

80 adult schizophrenics of either sex were randomized to receive standard doses of the two drugs orally, in a single blind manner, for 12 weeks. Interim follow-up visits were scheduled at 4 and 8 weeks. Effectiveness was assessed by changes in scores on the Brief Psychiatric Rating Scale (BPRS), Scale for Assessment of Negative Symptoms (SANS), Scale for Assessment of Positive Symptoms (SAPS) and physician administered Clinical Global Impression (CGI) scale. Tolerability was assessed by treatment emergent adverse events.

**Results:**

39 patients on amisulpride and 38 on olanzapine were evaluable. The groups were comparable at baseline with respect to age, sex, duration of illness and the rating scores. Final BPRS score at 12 weeks was significantly less for olanzapine (mean ± SD, 33.1 ± 9.79) than for amisulpride (38.2 ± 9.57). Olanzapine also reduced the positive and negative symptom scores better. Adverse drug reactions (ADRs) were encountered in 69.23% and 54.29% patients (p = 0.342) on amisulpride and olanzapine respectively. The incidence of tremor and insomnia were greater with amisulpride, while weight gain and sedation was more with olanzapine. No serious ADRs were encountered.

**Conclusion:**

Although the incidence of ADRs is comparable, olanzapine appears to have greater effectiveness in controlling symptoms of schizophrenia than amisulpride. ADR profiles show some difference.

**045**

**Cytotoxic activity of methanolic extract of *Berberis aristata D.C.***

Das S, Mathur R

**Delhi Institute of Pharmaceutical Sciences and Research, New Delhi, India.**

Natural products represent a reservoir of diverse templates and are being tapped to outsource novel anticancer agents. *Berberis aristata D.C.(Fam: Berberidaceae)* has been reported to be useful for ENT infections, indigestion, uterine and vaginal disorders. In the present study, the methanolic extract of the stem of *Berberis aristata*, was investigated against human breast cancer cell line (MCF7) to explore its anticancer potential. The effect of *Berberis aristata* methanolic extract (BAME) on proliferation of MCF7 cancer cell line was determined by microculture tetrazolium assay (MTT). The cells were exposed to different concentrations (100, 50, 25, 12.5, 6.25, 3.125 and1.5 *µ*g/ml) of BAME or vehicle for 48 h. Cisplatin (5, 2.5 and 1.25 *µ*g/ml) acted as positive control and vehicle (DMSO) as negative control. Following treatment, the cells were exposed to Tetrazolium dye (5mg/ml) for 4 h. The formation of the purple coloured formazan complex was dissolved by adding DMSO (100 *µ*l) and read at490nm using ELISA microtiter plate reader to determine the inhibitory concentration, IC_50_. About 40% increment in cell killing was seen when the dose of BAME was increased from 1.5 to 25 *µ*g/ml. At a concentration of 100 *µ*g/ml, 49.81% cytotoxicity was recorded. The IC_50_ value of BAME was 1.8964 *µ*g/ml after 48 h of incubation. In this study, it was observed that BAME induces a concentration dependent inhibition of MCF7 cells, with an IC_50_ value of 1.8964 *µ*g/ml after 48 h of incubation.

**046**

**Estimation of serum calcium and magnesium ratio in normal pregnancy, preeclampsia and eclampsia**

Kundu T, Paul S^1^, Bose M^1^, Dutta S

**Medical College, Kolkata, West Bengal, India.**

**Objective:**

Estimation of serum calcium and magnesium ratio in three trimesters of pregnancy, Preeclampsia and Eclampsia to determine whether its alteration has any role in maintenance of normal pregnancy and occurrence of pregnancy induced hypertension (PIH).

**Methods:**

It is a prospective cross-sectional study where serum calcium and magnesium level has been estimated in 148 women (15-35 years) in four different groups (Non-pregnant control, normal pregnancy in three trimesters, Preeclampsia and Eclampsia) by chemical assay using O-Cresolphthalein complexone method and Arsenazo magnesium reagent in EBRA Test Kit respectively. Reading was taken in a spectrophotometer & statistical analysis done by Student’s unpaired T test.

**Results:**

(i) There is a significant lowering of serum calcium and magnesium in pregnancy progressively decreasing with increasing gestational age in comparison to the control group and in preeclampsia and eclampsia when compared to third trimester of gestation (ii) Though the difference in serum calcium level between preeclampsia and eclampsia was not statistically significant, the same was statistically significant for magnesium. (iii) Serum calcium and magnesium ratio increased proportionately with increasing gestational age, in preeclampsia and eclampsia.

**Conclusion:**

The level of both the essential ions is found to fall progressively but the fall in magnesium being more than calcium, their ratio steadily increases with advancement of pregnancy, preeclampsia and eclampsia. Routine estimation of these ions may be useful as a diagnostic marker in high risk pregnancies and supplementation of calcium and magnesium to all pregnant mothers may reduce the risk of developing preeclampsia.

**047**

**Prescription patterns of drugs in patients with ischemic cerebrovascular disease in tertiary care hospital**

R Priyalatha, HV Anuradha, MC Shivamurthy

**M.S.Ramaiah Medical College and Teaching Hospital, Bangalore, India.**

**Objective:**

To analyze the drug prescribing patterns among inpatients admitted with ischemic crebrovascular disease in the neurology ward.

**Methods:**

The study was carried out over three month’s period (May 1, 2008 to July 31, 2008) at Ramaiah Memorial Hospital, a tertiary care hospital in Bangalore. The case records of patients discharged from the neurology ward during the study period were analysed. The study sample included 63 patients. Descriptive statistical method was used to analyze the data.

**Results:**

63 patients were admitted during the study period, of which 39 (61.9%) were males. The mean duration of hospitalizatlon was 7.3 days. The mean number of drugs prescribed per admission was 8.3 and 99.5% of the drugs were prescribed by brand names. Dyslipidemia (85%), hypertension (57%), diabetes mellitus (35%), were the associated conditions. Drug prescription patterns among inpatients could be grouped into antiplatelets, antihypertensives, hypolipidemics, hypoglycemic agents, antiepileptics and diuretics. Most commonly used antiplatelet drugs were aspirin (58.7%), clopidogrel (58.7%), combination of aspirin and clopidogrel (23.8%), Enoxaparin (57%). Antihypertensives were amlodipine (30%), Perindopril (19.6%), atenolol (14.2%). Hypolipidemic agents were atorvastatin (86%), fenofibrate (36.5%). Most commonly used hypoglycemic agents were Insulin (34.9%) and metformin (21%). Frusemide (16%) and mannitol (6.3%) were the diuretics used and Phenytoin sodium (16%) was the Antiepileptic of choice. Citicholine (35%), pantaprozole (55%), multivitamin (17.5%) were the miscellaneous drugs used. Enoxaparin, atorvastatin, citicholine and pantaprozole were not included in the WHO essential drug list.

**Conclusion:**

Antiplatelets aspirin (58.7%), clopidogrel (58.7%), antihypolipedemics atorvastatin (86%) were the commonly used drugs.

**048**

***In vitro* antisickling activity of *Zingiber officinale***

Kshatriya AA, Patel MD, Jadhav SR, Saraf MN

**Bombay college of Pharmacy, Mumbai, India.**

**Methods:**

The *in vitro* antisickling activity was evaluated using Emmel’s test protocol and sodium metabisulphite induced sickling on blood samples collected from anemic patient (sickle cell anemia). Briefly, the sickle blood samples were incubated with saline and test agent for 5 mins respectively. In Emmel’s test, anaerobic conditions were developed by applying vacuum to the blood samples at 37°c for 24h. In Sodium metabisulphite induced sickling, blood samples were incubated with sodium metabisulphite (2% w/v) for 20 mins at 37°c. The effect of drug on parameters such as ATPase, Heinz bodies, Met-Hemoglobin formation, Hemolysis of HbSS erythrocytes and HbS polymerization due to sickling was evaluated. The pharmacokinetic profile of drug action with respect to onset and duration of action, complete blood count was evaluated. The results were expressed as Mean ± SEM. Students’t test was applied and *P*<0.05 was considered as statistically significant.

**Results:**

*Zingiber officinale* significantly exhibited 71.4 ± 1.64% and 64.3 ± 1.64% inhibition of sickling at concentration of 100*µ*g/ml following Emmel’s test protocol and sodium metabisulphite induced sickling respectively.

**Conclusion:**

The present investigation revealed potential usefulness of *Zingiber officinale* could be in treatment of sickle cell anemia. The mechanism of action could be inhibition of HbS polymerization and blockage of Na^+^ and Ca^2+^ channels and opening of Mg^2+^ channels.

**049**

**Centchroman potentiates mitochondrial events leading to oxidative insult in human breast cancer cells**

Ranjan V^1^, Nigam M^1^, Zaidi D^1^, Sharma R^1^, Sundaram S^2^, Balapure AK^1^

**^1^Central Drug Research Institute, Lucknow, ^2^University of Allahabad, Allahabad, India.**

Centchroman (CC) has a proven antineoplastic action in MCF-7 and MDA MB-231 Human Breast Cancer Cells (HBCC) established by us and is undergoing phase III clinical trials. Apoptosis in conjunction with oxidative stress accomplishes in CC induced cytotoxicity HBCCs. Oxidative stress is a key factor involved in drug-induced apoptosis. We have explored the basis of oxidative stress mediated antineoplasticity of CC in these cell types using Tamoxifen (TAM) as a positive control. Role of Reactive Oxygen Species (ROS) and antioxidant enzymes like Superoxide dismutase (CuZn-/Mn-SOD), Catalase (CAT), Glutathione transferase (GST) and total Glutathione (GSH) has been investigated rendering oxidative stress involved in drug induced cytotoxicity. ROS was quantified using DCFDA dye qualitatively and quantitatively through fluorescence microscopy and FACS respectively. Immunoblotting was performed to assess the role of CuZn-/Mn-SOD, CAT, GST and GSH according to reported procedures. ROS generation peaks at 1micromolar declining further in a dose-dependent fashion depicting mitochondrial events involved. Time-dependent kinetics at 1micromolar depicts ROS generation as an early event involved. There is an increase in CuZn-SOD and GST while decrease in Mn-SOD and unaltered CAT expression suggesting oxidative stress. Decrease in total GSH signifies decreased antioxidant defenses thereby inducing cell death. Results indicate implication of critical mitochondrial events in CC induced apoptosis. This also confirms that antineoplastic action of CC in both the cell types involves extrinsic and intrinsic pathways of apoptosis along with oxidative stress. Exposure of cancer cells to ROS generating anticancer agents exhausts the cellular antioxidant capacity thus leading to apoptosis.

**050**

**Predictors of glomerular filtration rate in young, healthy Indian males**

Ullal SD, S Rajeshwari, Adhikari P, Pai MRSM

**Kasturba Medical College, Mangalore- 575001, India.**

**Background:**

Knowledge of the renal function of a patient is important to individualize drug therapy and drug dosage. Serum creatinine concentration is widely used as an index of renal function, but this concentration is affected by factors other than glomerular filtration rate (GFR). Hence, renal function is typically estimated by using certain specifically derived prediction equations. Two formulae have been most widely used- the Cockcroft-Gault (CG) formula and the more recent, Modification of Diet in Renal Disease (MDRD) study equation. Though both the formulae have been validated in Western populations, there is still a need to validate them in Asian populations. This study compares these two - the CG formula and the MDRD study equation, in young, healthy Indian males.

**Methods:**

Three hundred healthy male patients below the age of 60 years were enrolled. Demographic characteristics, serum creatinine and blood urea were recorded. Creatinine clearance was estimated by the CG formula and the MDRD study equation. Estimated creatinine clearance obtained by the two methods was compared using student’s t test.

**Results:**

Mean age of the study group was 23.8 years. The mean serum creatinine was 1.09mg/dL. Student’s t-test did not show any significant difference between the estimated GFR by CG formula and MDRD study equation (*P*=0.3102).

**Conclusion:**

In young healthy individuals, with serum creatinine within normal limits, both the CG formula as well as the MDRD study equation estimated similar GFR values. Hence in this study population either of the two formulae tested could be used to estimate GFR.

**051**

**A comparative assessment of efficacy and safety of oral antidiabetic drug combinations, metformin plus pioglitazone versus metformin plus glimepiride in type-2 diabetes mellitus**

Biswas R K, Paul K, Mukherjee A K, Bhattacherjee I, Mondal S

**R.G.Kar Medical College, Kolkata- 700004, India.**

**Introduction:**

In developed and developing nations there is explosive increase in prevalence of DM. As metformin prevents macro-vascular complications, sensitizes insulin and decreases appetite, it is combined with other group of drugs i.e. pioglitazone and glimepiride to maximize therapeutic efficacy and minimize toxicity.

**Methods:**

The protocol was placed to IEC and permission was taken. It was a randomized, single-blind parallel study involving 60 patients of both sexes. Group-1(n=30) received pioglitazone and metformin and group-2(n=30) received glimepiride and metformin. Total treatment period was 12 weeks. Fasting and PP BS level were examined at baseline, two follow-up visits and at the end of study, (at 4 weeks intervals). HbA1C, LFT, lipid profile and RFT were done at baseline and study-end. Statistical analysis were done by paired and unpaired ‘t’ tests.

**Results:**

Both group-1and 2 produced very effective glycemic control in fasting and PP BS and HbA1C l (*P* < 0.0001) without any hypoglycemic effect. Group-1 achieved better control of fasting BS (*P* = 0.01), significantly lowered triglyceride (*P* = 0.02) and increased HDL (*P* < 0.0001). Group-1 caused increase in SGOT (*P* = 0.001), SGPT (*P* = 0.01), body weight (*P* = 0.0002) and BMI (*P* = 0.0003).

**Conclusion:**

Though group-1 appears to be favourable, as it controls fasting BS better and is expected to prevent CVS complication of DM, its not possible to favour a particular regimen just now. It requires a prolonged study in larger population to delineate long-term effect on glycemic control, safety and prevention of immediate and late complications of DM.

**052**

**A random survey of prescription pattern of oral antidiabetics in patients of type II diabetes mellitus in a tertiary care teaching institute of North India**

Bedi S, Goel AK, Rai J, Kumar H

**Government Medical College, Amritsar, India.**

Diabetes mellitus is a multisystem disorder affecting millions globally. Multiple types of oral hypoglycaemic agents are available with variable prescription patterns. The aim of present survey is to know about pattern of prescription of oral antidiabetic agents in Type II Diabetic mellitus patients. 100 prescriptions of type II Diabetes mellitus patients were collected randomly from outpatient department and Diabetic clinic from Department of Medicine, Guru Nanak Dev Hospital. Patients were analysed for monotherapy and combination therapy. 52% were males and 48% were females in the age range of 39- 66 years. 66% patients were on combination therapy and 34% were on monotherapy. Glimepiride and metformin combination was prescribed to 28% patients. Gliclazide and metformin was prescribed to 14% patients. Triple drug combination including glimepiride, metformin and pioglitazone was prescribed to 8% patients. Glimepiride as monotherapy was prescribed to 14% patients; metformin was prescribed to 9% patients. The pattern shows an increasing use of glimepiride both as monotherapy as well as combination therapy.

**053**

**Plasma level monitoring of mycophenolic acid in renal transplant recipients**

Sarangi SC^1^, KH Reeta^1^, Agarwal SK^2^, Guleria S^3^, Gupta YK^1^

**Departments of ^1^Pharmacology; ^2^Nephrology; ^3^Surgery, All India Institute of Medical Sciences, New Delhi – 110029, India.**

**Background:**

Use of mycophenolic acid (MPA) in renal transplant recipients is increasing. Though it is known to cause dose and/ or concentration related gastrointestinal and haematological side effects, there is no optimal dosing regimen. The aim of the present study was to estimate plasma level of MPA and to correlate it with clinical outcomes.

**Methods:**

In this 3 month prospective trial, MPA plasma level and its AUC were estimated by limited sampling strategy (2h, 4h and 9h sampling time) using HPLC method in 25 renal transplant recipients receiving MPA with tacrolimus. There was one drop out. The subjects were on a fixed dose regimen of MPA (mycophenolate mofetil 0.5- 1 g or enteric coated mycophenolate sodium 360- 720 mg twice daily). Acute graft dysfunction (AGD) episodes and adverse drug events (ADE) were assessed using laboratory data, clinical examination, Gastrointestinal Symptom Rating Scale (GSRS) and Gastrointestinal Quality of Life Index (GIQLI) (Kleinman *et al*., 2006).

**Results:**

There was wide inter-individual variation in plasma level and AUC of MPA. The subjects in lower MPA AUC (<30 mg.h/L) had significantly higher incidence of AGD (*P*= 0.034). The incidence of GI ADE [diarrhea (*P*= 0.031), acidity (*P*= 0.011)] significantly increased with the rise in MPA AUC levels. The overall GSRS score was significantly worse in higher MPA AUC (>60 mg.h/L) range specifically with diarrhea and reflux disease subscale scores, though there was no significant difference in GIQLI scores.

**Conclusion:**

This study suggests the potential of therapeutic drug monitoring of MPA with limited sampling strategy in improving clinical efficacy of MPA.

**054**

**Comparative study of chlorpromazine and resperidone on positive and negative symptoms of schizophrenia**

Gupta RK, Singam AP

**Mahatma Gandhi Institute of Medical Sciences, Sewagram, Dist. Wardha, India.**

**Introduction:**

Schizophrenia is a devastating mental disease that affecting human population worldwide with prevalence of about 1%. Typical and atypical antipsychotics are mainly used to treat schizophrenia. The typical antipsychotic have autonomic side effects and EPS but the drugs are cheaper and was found to more effective in treating positive symptoms. Atypical antipsychotic takes care of both positive and negative symptoms. Still there is need for research in pharmacological intervention to treat symptoms of the disease both positive and negative. Therefore it was decided to conduct the study to assess the efficacy of typical (chlorpromazine)l and atypical antipsychotic (Resperidone).

**Materials and Methods:**

It was a longitudinal single blind prospective study 100 patients attending Psychiatry OPD at KHS Sevagram, with Schizophrenia were selected (50 receiving Chlorpromazine and 50 receiving Risperidone).They were interviewed and were administered test drug and were followed up every 3 monthly for 1 year. Scoring was done according to PANSS (Positive and Negative Symptom Scale for Schizophrenia).

**Results:**

Typical antipsychotics are better to treat positive symptoms but not much effective in treating negative symptoms of schizophrenia. Atypical antipsychotics take care of both positive and negative symptoms. Compliance is better with atypicals.

**Discussion and Conclusion:**

After assessing the patient, whether he has predominant positive or negative symptoms the psychiatrist can decide either of typical or atypical antipsychotic and this might be helpful for the better treatment of the patient.

**055**

**Natural course of autoimmune thyroiditis in type 2 diabetes: Association with gender, age, diabetes duration and dyslipidemia**

Thakkar NV, Jain SM

**Dept. of Pharmacology, L.M. College of Pharmacy, Ahmedabad, India.**

**Aim:**

To investigate the natural history of autoimmune thyroiditis (AIT) in female patients with type 2 diabetes mellitus.

**Methods:**

Since July 2007, annual screening for thyroid disease has been performed in type 2 diabetic patients. Serum HbA1c, LDL and TSH levels were measured in 109 female diabetic patients (53 on oral hypoglycemic agents and 56 on Insulin therapy) and 56 hypothyroid patients against 50 normal female subjects. Anthropometric association and effect of drug therapy was also taken into the consideration.

**Results:**

Serum HbA1c levels were found to high (*P*<0.1) when DM subjects (8.57+0.58) and DH subjects (8.55+0.49) were compared to HT (5.53+0.2) and normal subjects (5.2+0.36). LDL levels were found high (*P*<0.1) when HT subjects (124.71+7.36) were compared with DH (88.73+8.99) and normal subjects (85.16+4.05). Serum TSH levels were also significantly higher in HT patients (9.83+1.76) when compared to DH subjects (4.13+0.19) and DM subjects (2.19+0.24). Therapeutically, the subjects of DH were found to be extremely stable at *P*<0.001 when compared with HT subjects.

**Conclusion:**

These two metabolic disorders are strongly co-related such that both help to improve the symptoms of one another. DM-diabetes mellitus, DH-diabetic hypothyroid, HT-hypothyroid

**056**

**Clinical presentation, chemotherapy regimen used and response to treatment in patients with acute myeloid leukemia in a tertiary care hospital**

Nandeesh BR, Reeta KH, Kumar Lalit, Gupta YK

**All India Institute of Medical Sciences, New Delhi, India.**

**Introduction:**

The present study evaluates clinical presentation, chemotherapy regimen used and response to treatment in patients with acute myeloid leukemia.

**Methods:**

Retrospective study was done using data of patients treated at AIIMS.

**Results:**

Out of 12 patients, 9 were in age group of 20-40, 1of 17 years, 2 > 50 years. Most patients presented with fever (9), fatigue (8), loss of appetite (7) while few presented with bleeding from nose and gums (2), swelling of orbit and maxillary sinusitis (1), herpetic lesion (1), erythematous maculo-papular rash (1). 2 patients had prior myelodysplasia. 5 patients had hepatomegaly, 2 had splenomegaly and 1 had cervical lymph node enlargement. In most patients no cardiovascular, respiratory, neurological, hepatic or renal co-morbid conditions were present. 8 patients had hemoglobin less than 6 g/dL, 4 patients had platelet count less than 30, 000/mm^3^. Five patients had M2, three M3, two M4 and one M5 sub type AML (1 patient did not turn up for further investigations and treatment). 7 patients received cytarabine and daunorubicin, 1 patient had received mitoxantrone as induction chemotherapy, 1 patient didn’t turn-up for treatment. 6 patients developed febrile neutropenia during induction (1 had central line infection). 5 received 3 cycles of cytarabine consolidation chemotherapy, 2 received 2 cycles, and 1 patient received 1 cycle of cytarabine. 3 patients with M3 subtype received all-trans-retinoic acid.

**Conclusion:**

Most patients with acute myeloid leukemia in this study were in 20-40 year age group with out any associated co-morbid conditions and they well tolerated chemotherapy.

**057**

**Headache: A real “headache” for medical students**

Arora S, Chopra SC, Singh T

**Dayanand Medical College and Hospital, Ludhiana, India.**

Headache is a common medical complaint among medical students which affects the quality of life to a great extent due to limitation of daily activities. This project was undertaken to study the frequency of headaches among medical students and the pattern of medication followed. The study was carried out in 113 medical students (56 males and 57 females), aged 18-25 years, using a semi-structured questionnaire. A majority of respondents (53.9%) had 1-2 episodes of headache during the preceding 3 months whereas 23% had 3-4 and 17.7% had 5 or more episodes. Only 7 students (6 males and 1 female) had no reported episode of headache during the preceding 3 months. Triggering factors were reported by 65.5% cases, the major ones being mental (40.7%) and physical stress (18.6%). Nearly one fourth of the respondents had a positive family history for similar headaches. 72.6% of the respondents got relief by rest and 15.9% by head massage. Only 7 respondents had sought medical attention while the others relied on self medication or prescription by a friend or relative. Out of those who took NSAIDs as analgesics, 79.4% used a single drug, majority being Paracetamol (47%) or Nimesulide (22.7%), and 20.6% used a combination of NSAIDs, out of which 70.6% used Ibuprofen+Paracetamol. Only three respondents were on specific drugs for migraine, including ergotamine and propranolol. A majority of medical students are always under physical and mental stress which can result in headaches for which most indulge in self medication.

**058**

**Comparative, randomized, double blind, parallel, multi-centric study of efficacy and safety of pregabalin + methylcobalamin versus gamma linolenic acid + methylcobalamin in patients with diabetic peripheral neuropathy**

JC Shobha, Sagar Vidya, Ramadoss K, Sitaramaih BS, Sankar K Ravi, Sridhar PVB, Chary NVS, Balamurugan, Bakthavatsalam, Shanmugasundram R, Senthivel N, Jayachandran K, Reddy B Mohan

**Department of Clinical Pharmacology and Therapeutics, Nizam’s Institute of Medical Sciences, Punjagutta – Hyderabad. A.P., India**

**Objective:**

To compare the clinical efficacy and safety of Pregabalin 75mg + methylcobalamin 750*µ*g versus Gamma linolenic acid 120mg + methylcobalamin 500*µ*g in patients with diabetic peripheral neuropathy.

**Methods:**

Demographic and clinical data like Total pain rating index (TOPRI); present pain intensity by VAS (PPI - VAS); overall intensity of total pain experience, rating scales for diabetic peripheral neuropathy (SF-McGill Pain Questionaire) were recorded at baseline, 4 and 8 weeks. Investigations were done at baseline and at the end of the study. Safety evaluation was done at each follow up visit. Global evaluation by the patient and as well as the investigator was done at the end of the study.

**Results:**

231 patients were enrolled, 197 patients completed the study. In the pregabalin + methylcobalamin group (n = 106 (M73:F33)), as compared to baseline there was significant decrease in TOPRI from 15.4 ± 13.4 (baseline) to 2.6 ± 2.6 (8weeks) (*P*< 0.05). Similarly, there was significant reduction in PPI – VAS from 66.1 ± 16.4 (baseline) to 18.6 ± 8.9 (8weeks) (*P*<0.05) and overall intensity of total pain experience was 2.8 ± 1.1 (baseline) to 0.79 ± 0.5 (8weeks) (*P*<0.05) as measured by SF Mc Gill pain questionnaire. In the Gamma linolenic acid+ methylcobalamin group (n= 91 (M61:F30)), as compared to baseline there was significant decrease in TOPRI from 14.2 ± 12.1 (baseline) to 3.4 ± 4.2 (8 weeks) (*P*< 0.05). Similarly, there was significant reduction in PPI – VAS from 64.2 ± 14.0 (baseline) to 25.9 ±12.7 (8weeks) (*P*<0.05) and overall intensity of total pain experience was 2.6 ± 0.9 (baseline) to 1.0 ± 0.6 (8weeks) (*P*<0.05) as measured by SF Mc Gill pain questionnaire. At the end of 8 weeks as compared to gamma linolenic acid + methylcobalamin, pregabalin + methylcobalamin showed statistically significant improvement in PPI – VAS and overall intensity of total pain experience.

**Conclusions:**

Combination of Pregabalin 75mg + methylcobalamin 750*µ*g in twice daily dose can be safely be used in the management of diabetic peripheral neuropathy.

**059**

**Challenges in bioequivalence of highly variable drugs**

Mehta H^1^, Shah S^1^, Velu T^1^, Srivastava A^1^, Vikas Raj^2^

**^1^Torrent Research Centre, Torrent Pharmaceuticals Ltd., Gandhinagar, ^2^Cadila Pharmaceuticals Ltd., Ahmedabad, India.**

**Purpose:**

To demonstrate how replicate design is a better approach to solve the bioequivalence challenge of highly variable drug with respect to crossover design by using simulation (two compartmental model) i.e. generating data for 1000 subjects based on the macro and micro constants obtained from the in-vivo data.

**Materials:**

Mean concentration of Propafenone HCl at different time points was determined in plasma using one compartment model with log-normal statistical distributions of intersubject and intrasubject variability in pharmacokinetic parameters. By using mean concentration, 1000 plasma concentrations were simulated (with the 30% variability) with the help of WinNonlin^®^ software. Crossover study was carried out (by random selection of n = 48 plasma concentrations) for 24 subjects and replicate study was carried out (by random selection of n = 96 plasma concentrations) for 24 subjects.

**Data and Results:**

Intra-subject C.V. for ln (C_max_), ln (AUCt) and ln (AUC_Inf_) was reduced from 27% to 10%, from 32% to 15% and from 33% to 16% respectively in replicate design compared to crossover design. For cross-over and replicate design the 90% C.I. for C_max_ was 80.30-116.16 and 88.6-117, for AUC_t_ it was 74.79- 115.46 and 77.7-120.10 and for AUC_Inf_ it was 71.80-112.59 and 77.7- 120.12 respectively.

**Conclusion:**

Result shows that replicate designs for highly variable drug effectively eliminate the intra-subject variability with respect to crossover design. Replicate design also condensed the 90 % C.I. limit for C_max,_ but did not show considerable reduction in the AUC_t_ and AUC_Inf_.

**060**

**Institutional ethics committees in India: Need for capacity building and enhanced oversight to improve protection of participants**

Gupta Madhur, Gupta YK

**Department of Pharmacology, All India Institute of Medical Sciences, New Delhi-110029, India.**

**Objective:**

Initiatives to streamline drug discovery and development research in the country have brought about changes in the regulatory landscape in India. The objective was to assess the status of regulatory and GCP guidelines compliance of Institutional and Independent Ethics Committees in India.

**Method:**

This observational study, by a validated questionnaire, was designed to assess the status of Ethics Committees in India, their operational functioning, standard operating procedures, oversight in multicentre trials, special populations, and compliance of good clinical practices, informed consent process, and Schedule Y.

**Results:**

The questionnaire was completed by members or clinical investigators of 64 Institutional Ethics Committees in India (primarily medical and pharmacy colleges). Though most ethics committees function by written standard operating procedures, several issues like the need of GCP training to members, constraints in funding, publication of research, media handling, and oversight in multicenter trials need to be strengthened.

**Conclusion:**

Registration of ethics committees, capacity building, and harmonization of Ethics Committees and their SOP’s are the major thrust areas to enhance regulatory compliance. There is a need to modulate the concept and processes to ensure a higher degree of protection to clinical trial participants

**061**

**A comparitive study of efficacy and tolerability of ibuprofen vs aceclofenac in osteoarthritis of knee joint**

Klair J

**Govt. Medical College, Patiala (Punjab), India.**

A randomized single blind parallel group comparative study of 50 patients of osteoarthritis of knee fulfilling the inclusion criteria was done. After verifying the exclusion criteria patients were divided into 2 groups of 25 each after a written informed consent. 25 patients were prescribed 400mg tds Ibuprofen and 25 patients were put on Aceclofenac 200mg/day for 6 weeks. At the end, results of the study comparing the efficacy and tolerability of the Ibuprofen with Aceclofenac were statistically analyzed. This study comprised of 50 patients of osteoarthritis coming to the out patient department (OPD) of Orthopaedic department of Government Medical College and Rajindra Hospital, Patiala which is divided into two groups. A detailed history and physical examination was carried out in these patients. Patients of either sex (age 30-75 years) with confirmed diagnosis of osteoarthritis of knee joint of more than 3 months duration and fulfilling inclusion criteria were considered. Eligible patients were divided equally and randomly into two treatment groups. Group A patients were given Aceclofenac and Group B patients were given Ibuprofen. The problems related to osteoarthritis (OA) knee and its management is expected to increase with rise in the proportion of elderly population. Therefore, it was important to study the effect of treatment on patients suffering from a chronic condition such as OA of knee.

**062**

**Efficacy of sodium valproate and haloperidol in the management of acute mania. A comparative study**

Kalra BS^1^, Sekhar S^1^, Mendhekar DN^2^, Tekur U^1^

**^1^Maulana Azad Medical College, ^2^G.B.Pant Hospital, Delhi, India.**

**Objective:**

The study was carried out to compare the efficacy of intravenous sodium valproate with intramuscular haloperidol in patients of acute mania.

**Method:**

A total of thirty patients meeting DSM-IV criteria for acute manic episodes were enrolled for the study. They were further divided into 2 groups of 15 patients each. One group was treated with haloperidol (10mg, intramuscular) twice daily while the other group received sodium valproate (500mg, intravenous) twice daily. The patients were assessed on each day from day 1 to day 5 and then on day 9 and day 13. Improvement in symptoms was assessed by reduction in Young Mania Rating Scale (YMRS). Outcome criterions for analysis were latency of response and remission; any additional drugs required for sedation; duration of stay in the hospital.

**Results:**

At the end of 2 week study period, overall response rate in both the groups were similar (*P*>0.1). In comparison to haloperidol group, patients treated with sodium valproate showed faster response, on day 5 significant reduction in YMRS score was observed in the group treated with sodium valproate (*P*<0.05). Total amount of lorazepam as additional sedative was less in patients treated with sodium valproate. Extrapyramidal episodes were observed in 60% of patients treated with haloperidol. There was however no difference in both the groups with regard to duration of stay in the hospital.

**Conclusion:**

Sodium valproate in the treatment of acute mania is as efficacious as haloperidol but provides faster response. It is relatively safer as compared to haloperidol. Further studies should be undertaken preferably double blind with large sample size to substantiate the observations.

**063**

**A study on steroids as adjuvant in the treatment of pyogenic meningitis in children in paediatric ward in tertiary care centre at Kanpur**

Khan S, Jain IP, Singh SP, Singh S

**G.S.V.M. Medical College, Kanpur, UP, India.**

**Objective:**

Assessment of the effectiveness of dexamethasone in management of acute bacterial meningitis in children.

**Materials and Methods:**

A prospective study was conducted in paediatric ward for the period of two months at LLR hospital Kanpur. In a double-blind controlled trial, we included 71 children aged below 15 years with pyogenic meningitis who had been admitted to the children’s ward. The primary outcome was overall death. Secondary outcomes included sequelae, in-hospital deaths.

**Inclusion Criteria:**

Children aged less than 15 years with pathological evidence of bacterial meningitis.

**Exclusion Criteria:**

Patients aged above15 years, viral meningitis, tubercular meningitis, fungal meningitis, space occupying lesion, subdural haemorrage.

**Results:**

Of the 71 included children belonging to age group <2 years: 20(29%), 2-5 years:34(48%), 5-15years:17(23%).37 (52%) were assigned to dexamethasone 0.15 mg / Kg /dose, 6 hourly for 48 hours and 34 (48%) to placebo. 41(58%) of 71 patients had *Streptococcus pneumoniae*, 22(32%) *Haemophilus influenzae* type b, 6(8.5%) Neisseria meningitidis and 2(1.4%) *Salmonella* spp. The number of overall deaths was the same in the two treatment groups. At final outcome, sequelae were identified in 16(23%) of children on steroids and in 22 (32%) on placebo.

**Conclusion:**

Study shows that Most of children belong to age group 2-5 years. Most common offending agent for meningitis was *Streptococcus pneumoniae*. Children receiving steroid had decreased in morbidity but overall mortality was same in both groups.

**064**

**A prospective study on prescribing pattern of drugs in pediatric ward of Kathmandu university teaching hospital**

Khan GM^1^, Jha AK^2^, Pokharel A^1^, Kumpakha A^1^, Regmi A^1^, Gochhe B^1^, Bajracharya^1^

**^1^Kathmandu University, Dhulikhel, Nepal; ^2^Dhulikhel Hospital, Dhulikhel, Kavre, Nepal.**

**Introduction:**

Infants and children are the most vulnerable population groups to contact illness. Antibiotic is commonly prescribed in pediatric patients. The key role of antibiotics for the treatment of infectious diseases.

**Objective:**

To obtain information on pediatric prescribing pattern of antibiotic in Nepal.

**Methods:**

This prospective study was performed in the pediatric ward.

**Results:**

Total number of 105 patients was taken for the study 89 from general ward and 16 from NICU. The disease of respiratory system (39.33%) and hyperbilirubinemia (68.75%) were predominant in general ward and NICU respectively. 53.93% of general ward patients received antibiotic where penicillin was most prescribed drugs, 75% were given only one antibiotic. Penicillin and aminoglycoside were most prescribed antibiotic in neonates. 56.25% of the total antibiotics were administered orally in ward patient and all antibiotics were administered parenterally in neonates. NSAIDs prescribed 55.05% of general ward patients. 54.15% of drugs prescribed by generic name and 70.59% of prescribed drugs from EDL. Significant difference found between age group of patient and disease encountered (χ^2^=36.6, *P*=0.05). There was significant difference between diseases of different system with respect to cost (F=7.69, *P*=0.01).

**Conclusion:**

While prescribing drugs in pediatric, the main challenges are choosing the right drug for them, hence physician should have good knowledge on choice of drug regimen in clinical situation, should emphasize on laboratory investigation for diagnosis and prescribing cost effective drugs. Also the increase in qualified and trained health worker like increase in hospital pharmacist would be helpful for the rational use of drugs.

**065**

**Drug utilization study in a trauma care unit of a tertiary care hospital**

Mariguddi D.D, Jeevanagi S.R, Kakkeri R.H, Patil B.V, S.Manjunath

**M.R.Medical College, Gulbarga, India.**

**Introduction:**

Trauma is major disease of modern mankind. The present study is to assess prescribing trends in a trauma care unit of a tertiary care hospital in Gulbarga City.

**Methods:**

A prospective cross-sectional study was conducted for 15 months in Basaweshwar Teaching and General Hospital Gulbarga. The data were analysed using WHO core indicators like prescribing indicators and patient care indicators.

**Results:**

A total of 110 patients where interviewed and their prescriptions were studied. The average number of drugs per prescription was 3.5 to 9.5. 46.77% of drugs were from WHO essential drug list and 2% were generics. The prescribing frequency of Diclofenac sodium (97%) was more, when compared to other analgesics. Among the drugs used to control oedema, Intravenous mannitol (47.27%) and oral glycerol (7.27%) were used. 79.09% of patients were given injection tetanus toxoid for tetanus prophylaxis. 96.5% prescriptions contain antimicrobials. The availability of the drug in the hospital was satisfactory (98%). The average cost of drugs per day per patient was ranging from Rs. 600-750/-. The average time given for consultation per patient was 12.5 minutes.

**Conclusion:**

The incidence of polypharmacy and use of non-generic names were very high. Drugs prescribed from essential drug list was only 50%. The newer antimicrobial and newer proton pump inhibitors were prescribed more, but tetanus prophylaxis is not 100%. Prescription by generics should be promoted for costeffective treatment. The results indicate that there is a considerable scope for improving prescribing habits according to rational drug use and to provide a feed back to hospital authority.

**066**

**Efficacy and safety of antiretroviral therapy in prevention of vertical transmission of HIV infection**

Shashikala GH, Jayashree VN, Jyothi CH

**Department of Pharmacology, JJM Medical College, Davangere, Karnataka, India.**

**Objective:**

To study efficacy of HAART (Highly active antiretroviral therapy) in preventing vertical transmission of HIV infection and observe for adverse drug reactions.

**Methods:**

Retrospective and prospective studies were done between June 2005 and 2008 at Child Health Institute, Davangere. The study included 177 babies born to HIV+ve mothers. They were given prophylactic Nevirapine 2mg/kg at birth, Zidovudine 4mg/kg×4wks as per WHO protocol. If they came after birth only Zidovudine was given. ELISA done at 9 and 18 months of birth indicated HIV status. Maternal data was analysed for antenatal prophylaxis received and type of delivery. Babies were observed for adverse reactions.

**Results:**

Of 177 babies, 173(97.74%) were non-reactive and 4(2.26%) were reactive to ELISA. In reactive group, 2 babies had complete prophylaxis but mothers received no prophylaxis. In other 2 babies and mothers prophylaxis was incomplete All were vaginal deliveries with average birth weight 2.41 kgs. In non-reactive group, 132 babies(76.30%) and 78 mothers (45.08%) received complete prophylaxis. 45 (26.01%) were caesarian deliveries with average birth weight 2.62kgs. Adverse effects included skin rashes (4) anemia (2).

**Conclusion:**

HAART given as per WHO protocol has good efficacy which can be further improved with strict adherence to treatment. Incidence of adverse effects observed is also low.

**067**

**Prescribing pattern and cost-identification analysis of anti-infective use in respiratory tract infections in pediatrics department in a medical college hospital of North India**

Gupta K, Kaushal S, Chopra SC, Bains HS

**Dayanand Medical College and Hospital, Ludhiana, India.**

Respiratory tract infections are common in children requiring treatment with antimicrobial agents (AMAs). The aim was to identify prescribing pattern and cost-identification analysis of AMAs in respiratory tract infections in the pediatrics population in DMCH. The study was conducted over a period of two months (Nov-Dec, 2007) after approval by Institutional Ethics Committee. The data was collected daily in both Pediatric ward and ICU and entered in a structured proforma. Fifty nine patients were enrolled. The common diagnosis was bronchopneumonia (33.3%), bronchiolitis (17.5%) and bronchial asthma (7.9%). Average no. of AMAs received per patient was 3.9. The most common AMAs prescribed were cephalosporins 26.1%, aminoglycosides 20.9%, beta-lactams (except cephalosporins) 17.4%.and fluoroquinolones 11.1%.The group wise distribution shows that commonly prescribed AMAs were cefepime (35%), ceftriaxone (23.3%) and cefoperazone plus sulbactam (21.6%) among cephalosporins; amikacin (91.6%) among aminoglycosides; amoxicillin plus clavulanic acid (47.5%), cloxacillin (17.5%) and meropenem (15%) among beta-lactams (except cephalosporins). The preferred route of administration for AMAs was intravenous (86.1%), followed by oral (11.3%). Average cost of AMA per patient was Rs 6942. Beta-lactams contributed to 44% of the total AMA cost followed by cephalosporins (31.8%) and miscellaneous AMAs (8.5%). The outcome was favourable in 84.7% of the patients. DMCH being a major referral centre of the state, newer generation of AMAs are more commonly prescribed which leads to the increased cost. This preliminary study helped us to identify the pattern of AMA prescribing based on which future intervention studies may be planned to promote rational drug use.

**068**

**Correlation between umbilical cord and maternal intrapartum serum retinol in very low birth weight neonates**

Mishra S, Thomas K, Agarwal R, Gupta YK, Deorari AK, Paul VK

**All India Institute of Medical Sciences, New Delhi, India.**

**Introduction:**

Preterm very low birth weight (VLBW) neonates are known to be deficient in serum retinol at birth. There is paucity of data on vitamin A status of preterm neonates from India.

**Objectives:**

*Primary*: To evaluate the degree of correlation between maternal intrapartum (MI) and the umbilical cord (UC) serum retinol levels in preterm VLBW neonates. *Secondary*: To assess postnatal change in serum retinol level in these neonates at day-28 of age.

**Methods:**

Consecutively born 48 preterm VLBW neonates were enrolled in an institutional approved prospective study. Serum retinol was assayed by high performance liquid chromatography (HPLC). Enrolled neonates were followed till discharge/death/ or day-28 of life, whichever is later. Vitamin A intake of enrolled neonates was quantified and recorded prospectively till day-28 of age.

**Results:**

Average birth weight and gestation of the enrolled neonates was 1157±267 g and 31.6±2.7 weeks respectively. Mean vitamin A intake in first seven days and thereafter till day 28 was 1284±678 IU/day and 2475±930 IU/day respectively. Mean UC and MI serum retinol levels were 172.0±99.2 *µ*g/L and 443.6±295.6 *µ*g/L respectively. UC and MI serum retinol had a significant correlation (Correlation coefficient = 0.37; *P*=0.025). There was trend toward higher UC serum retinol levels in small for gestational age (SGA) compared to appropriate for gestational age (AGA) neonates (*µ*g/L; mean difference, 95%CI: 34.9, 90.6 to -20.8; *P*=0.21). Serum retinol levels on day-28 of age were significantly higher compared to UC (*µ*g/L; mean difference, 95% CI: 62.5, 14.4 to 110.5; *P* = 0.014).

**Conclusion:**

A significant correlation between UC and MI serum retinol was observed in preterm VLBW neonates. Serum retinol levels were significantly higher at 4 weeks of age.

